# A prosurvival DNA damage-induced cytoplasmic interferon response is mediated by end resection factors and is limited by Trex1

**DOI:** 10.1101/gad.289769.116

**Published:** 2017-02-15

**Authors:** Erkin Erdal, Syed Haider, Jan Rehwinkel, Adrian L. Harris, Peter J. McHugh

**Affiliations:** 1 Department of Oncology, Weatherall Institute of Molecular Medicine, University of Oxford, John Radcliffe Hospital, Oxford OX3 9DS, United Kingdom; 2 Medical Research Council Human Immunology Unit, Weatherall Institute of Molecular Medicine, University of Oxford, John Radcliffe Hospital, Oxford OX3 9DS, United Kingdom

**Keywords:** DNA damage, DNA repair, cancer, interferon, nucleases

## Abstract

Radiotherapy and chemotherapy are effective treatment methods for many types of cancer, but resistance is common. Recent findings indicate that antiviral type I interferon (IFN) signaling is induced by these treatments. However, the underlying mechanisms still need to be elucidated. Expression of a set of IFN-stimulated genes comprises an IFN-related DNA damage resistance signature (IRDS), which correlates strongly with resistance to radiotherapy and chemotherapy across different tumors. Classically, during viral infection, the presence of foreign DNA in the cytoplasm of host cells can initiate type I IFN signaling. Here, we demonstrate that DNA-damaging modalities used during cancer therapy lead to the release of ssDNA fragments from the cell nucleus into the cytosol, engaging this innate immune response. We found that the factors that control DNA end resection during double-strand break repair, including the Bloom syndrome (BLM) helicase and exonuclease 1 (EXO1), play a major role in generating these DNA fragments and that the cytoplasmic 3′–5′ exonuclease Trex1 is required for their degradation. Analysis of mRNA expression profiles in breast tumors demonstrates that those with lower Trex1 and higher BLM and EXO1 expression levels are associated with poor prognosis. Targeting BLM and EXO1 could therefore represent a novel approach for circumventing the IRDS produced in response to cancer therapeutics.

## Abstract (precis)

Here, McHugh and colleagues investigate the mechanisms by which antiviral type I interferon (IFN) signaling may be involved in resistance to radiotherapy and chemotherapy across different tumors. They demonstrate that DNA-damaging treatments used during cancer therapy lead to the release of ssDNA fragments from the cell nucleus into the cytosol, promoting an innate immune response. The factors that control DNA end resection during double-strand break repair, including the Bloom syndrome helicase (BLM) and exonuclease 1 (EXO1), play a major role in generating these DNA fragments, and the cytoplasmic 3′–5′ exonuclease Trex1 is required for their degradation.

The host innate immune system is essential in the defense against potentially lethal infections by a broad range of pathogens (Takeuchi and Akira 2010). It initiates the fight against the infection by direct recognition of molecular patterns, including DNA and RNA (Medzhitov and Janeway 2000; Janeway and Medzhitov 2002; Paludan and Bowie 2013; Orzalli and Knipe 2014). This immune system operates on the premise that no free endogenous DNA is present in the cytosol, and thus cytosolic DNA constitutes a molecular pattern of infection and is recognized as foreign (nonself) (Paludan and Bowie 2013; Orzalli and Knipe 2014). Virus-derived DNAs trigger innate immune signaling responses in infected host cells by the engagement of pattern recognition receptors (Kono and Rock 2008; Chen and Nunez 2010; Desmet and Ishii 2012). After the recognition of cytosolic DNA, cGAMP synthase (cGAS) activates STING via generation of 2′–5′ cyclic GMP–AMP (cGAMP) (Li et al. 2013; Sun et al. 2013; Wu et al. 2013; Wu and Chen 2014). STING in turn induces the phosphorylation and nuclear translocation of IFN (interferon) regulatory factors (IRFs) (Ishikawa et al. 2009). IRFs are transcription factors driving the transcription of several genes, in particular those encoding type I IFN. Type I IFNs play a central role in antiviral host defense and are key to an effective antiviral state by activating natural killer (NK) cells, NKT cells, and other immune cells (Billiau and Matthys 2009; Ivashkiv and Donlin 2014). The interaction of secreted type I IFN with its receptor leads to the activation of the JAK–STAT pathway. Activated JAKs phosphorylate STATs (signal transducers and activators of transcription), which form complexes that translocate to the nucleus and bind to IFN-stimulated response elements in DNA to initiate transcription of a variety of genes, so-called IFN-stimulated genes (ISGs) (Platanias 2005). Besides antiviral effects, it was shown that the expression of a set of IFN-induced genes leads to a so-called “IFN-related DNA damage resistance signature” (IRDS) (Weichselbaum et al. 2008). In breast cancer, IRDS expression measured by a clinical classifier comprised of seven IRDS genes (*STAT1*, *MX1*, *ISG15*, *OAS1*, *IFIT1*, *IFIT3*, and *IFI44*) identifies patients whose cancers are resistant to chemotherapy and radiotherapy. The silencing of IRDS genes resensitizes triple-negative breast cancer (TNBC) cells to chemotherapy and radiotherapy in vitro and in vivo, illustrating the potential therapeutic power of modulating this response (Boelens et al. 2014).

In this study, we show that self-DNA derived from DNA double-strand break (DSB) damage repair processes escapes the nucleus and triggers these signaling pathways. DSBs can arise from exposure to exogenous agents such as ionizing radiation (IR) or endogenously from attack by oxidizing reactive species and during the collapse of damaged replication forks (Wyman and Kanaar 2006). Two major pathways repair DSBs in mammalian cells: nonhomologous end-joining (NHEJ) and homologous recombination (HR). The former is operational throughout the cell cycle, while HR operates predominantly in cells that have passed through S phase, where the preferred sister chromatid substrate for the reaction is available (Symington and Gautier 2011; Chapman et al. 2012). HR is initiated through the generation of 3′ ssDNA overhangs that are the substrates for the downstream strand invasion steps of the process. Depending on the extent and nature of damage associated with the DSB termini, the nuclease activity of the Mre11 component of the Mre11–Rad50–NBS1 (MRX) complex is required to initiate resection, producing endonucleolytic incisions internally (5′) to the blocked end, providing a substrate for the 5′–3′ exonucleases that perform the long-range resection reactions (Symington and Gautier 2011; Cejka 2015). For DSB termini less affected by blocking lesions, the nuclease activity of Mre11 is dispensable (Llorente and Symington 2004), but the MRN complex plays a structural role in recruiting downstream resection factors. Two separate pathways have been identified that produce the 3′ overhang resection products of several thousand nucleotides that are required to promote HR in mitotic cells (Gravel et al. 2008; Mimitou and Symington 2008; Zhu et al. 2008; Cejka et al. 2010; Niu et al. 2010; Nimonkar et al. 2011). One of these pathways is dependent on a 5′-to-3′ exonuclease 1 (EXO1) acting in concert with the DNA-unwinding activity provided by the Bloom (BLM) helicase. The second pathway uses an alternative 5′–3′ nuclease activity present in DNA2, again in collaboration with the BLM helicase. Accordingly, cells lacking BLM and EXO1 are defective in both major long-range resection pathways (Symington and Gautier 2011; Cejka 2015).

Here, we show that DNA fragments generated during the long-range resection step of DSB repair accumulate in the cytosol and activate type I IFN signaling. We demonstrate that Trex1, the major cytoplasmic 3′-to-5′ DNA exonuclease in mammalian cells, which is anchored to the outer face of the nuclear membrane (Wolf et al. 2016), degrades IR-induced cytosolic DNA fragments and suppresses ISG expression. Moreover, we present evidence that expression of DNA repair nucleases required to generate the cytosolic DNA fragments plays an important prognostic role in breast cancer, validating them as attractive therapeutic targets to overcome resistance to DNA-damaging therapies.

## Results

### Therapeutic DNA-damaging agents induce the accumulation of cytosolic ssDNA

In pilot experiments, we observed that the treatment of mammalian cells with X-irradiation led to an accumulation of ssDNA in the cytosol, an observation consistent with several other studies that report that genomic stress is associated with the accumulation of ssDNA in the cytosol (Shen et al. 2015; Ho et al. 2016). To further investigate whether DNA damage processing can lead to DNA fragments escaping the nucleus, a range of breast cancer cell lines (MCF7, BT474, MDA-MB-231, HCC1806, and T47D) was treated with IR (X rays) or DNA-damaging chemotherapeutics (mitomycin C or cisplatin) that induce distinct forms of DNA damage. The major cytotoxic lesions produced by IR are DNA DSBs often associated with oxidative and chemical damage to the nucleotides at their termini (Lomax et al. 2013). In contrast, mitomycin C produces a mixture of DNA monoadducts and interstrand cross-links, while cisplatin induces monoadducts, intrastrand cross-links, and interstrand cross-links (McHugh et al. 2001). With both of these chemotherapeutics, the cross-links block replication fork progression, potentially triggering fork collapse events that are associated with the accumulation of toxic one-ended DNA DSBs (De Silva et al. 2000; McHugh et al. 2000).

**Figure 1.**
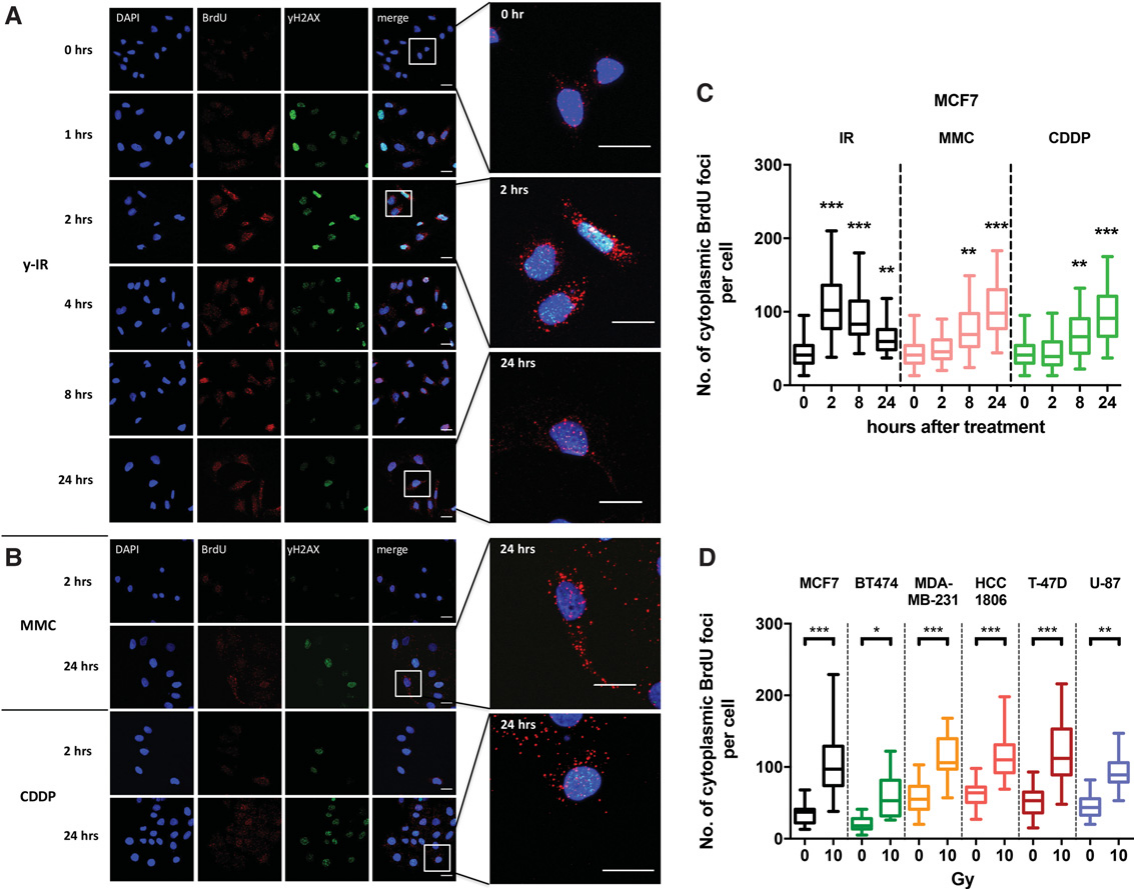
DNA-damaging agents increase the levels of cytosolic DNA. (*A*,*B*) Representative confocal images of MCF7 cells with BrdU preincorporated for 38 h (1.5 cell cycles); subjected to 10-Gy IR, 3 µM mitomycin C, or 15 µM cisplatin treatment; and followed microscopically for 24 h. Cells were stained for DNA (DAPI; blue), BrdU (red), and yH2AX (green). Bars, 20 µm. (*C*) Quantification of BrdU foci outside the nucleous per cell at the indicated time points following 10 Gy of IR, 3 µM mitomycin C, and 15 µM cisplatin. Boxes represent the upper and lower quartiles, the band represents the median, and the whiskers represent the minimum and maximum values in the data set. A minimum of 100 cells were scored per time point and grouped from three independent experiments. Statistical significance was determined by the Mann-Whitney *U*-test. (*D*) Quantification of BrdU foci outside the nucleous per cell in a panel of cancer cell lines 2 h following 10 Gy of IR. Boxes represent the upper and lower quartiles, the band represents the median, and the whiskers represent the minimum and maximum values in the data set. A minimum of 100 cells were scored per time point and grouped from three independent experiments. Statistical significance was determined by the Mann-Whitney *U*-test.

Cells were prelabeled with bromodeoxyuridine (BrdU) for 1.5 cell cycles and subsequently treated with either 10 Gy of IR, 3 µM mitomycin C, or 15 µM cisplatin. BrdU foci were visualized by immunofluorescence using an anti-BrdU antibody over the subsequent 24 h. In these experiments, we did not denature the cellular dsDNA; therefore, any BrdU foci observed correspond to labeled ssDNA. IR treatment significantly increased the number of γH2AX and BrdU foci in the nucleus at 2 h after treatment in MCF7 (Fig. 1A; Supplemental Fig. 1A), BT474, MDA-MB-231, HCC 1806, and T47D cells (Supplemental Fig. 1B), consistent with the induction of DSBs and subsequent DNA end resection in preparation for DSB repair. Strikingly, however, an accumulation of cytosolic BrdU foci was also evident. Elevated numbers of cytosolic BrdU foci persisted for >8 h, returning to near pretreatment levels at 24 h. Notably, the dynamics of the accumulation and resolution of the cytosolic DNA faithfully tracked that of the nuclear DNA BrdU foci. Moreover, substantially lower doses of IR (2 Gy and 6 Gy), the same as those applied clinically, were also effective in inducing cytosolic ssDNA (Supplemental Fig. 1C,D). Similar effects were seen after treatment with mitomycin C and cisplatin, although the accumulation of BrdU foci occurred later, between 8 and 24 h after treatment (Fig. 1B,C; Supplemental Fig. 2B,C), when cells were accumulating in the S and G2 phases of the cell cycle (Supplemental Fig. 2A).

The cell cycle distribution profile of the MCF7 cells was not affected by BrdU labeling, suggesting that the alteration in focus formation could not be explained by altered cell cycle progression in labeled cells (Supplemental Fig. 3). To confirm that BrdU-labeled foci represent the accumulation of ssDNA fragments, cells were treated with two mechanistically distinct ssDNA-selective nucleases, the S1 nuclease and P1 nuclease, both of which eliminated the staining (Supplemental Fig. 4A,B).

We quantified the difference in endogenous cytosolic ssDNA levels across different cell lines with TNBC cell lines (MDA-MB-231 and HCC1806) showing the highest numbers of ssDNA foci (Fig. 1D). MDA-MB-231 and HCC1806 cells also have correspondingly higher basal levels of DSBs as defined by γH2AX foci (Supplemental Fig. 5B). Nonetheless, a significant increase in cytosolic focus numbers after IR was seen in all cell lines tested, with MCF7 cells showing the greatest fold increase in cytosolic DNA accumulation after irradiation. Interestingly, the two cell lines with a higher basal level of DSBs and cytosolic DNA (MDA-MB-231 and HCC1806) are more radio-resistant than MCF7 cells, suggesting a potential protective effect of elevated cytosolic ssDNA at the cellular level (Supplemental Fig. 5C).

### DNA end resection factors are required for the accumulation of IR-induced cytosolic ssDNA

**Figure 2.**
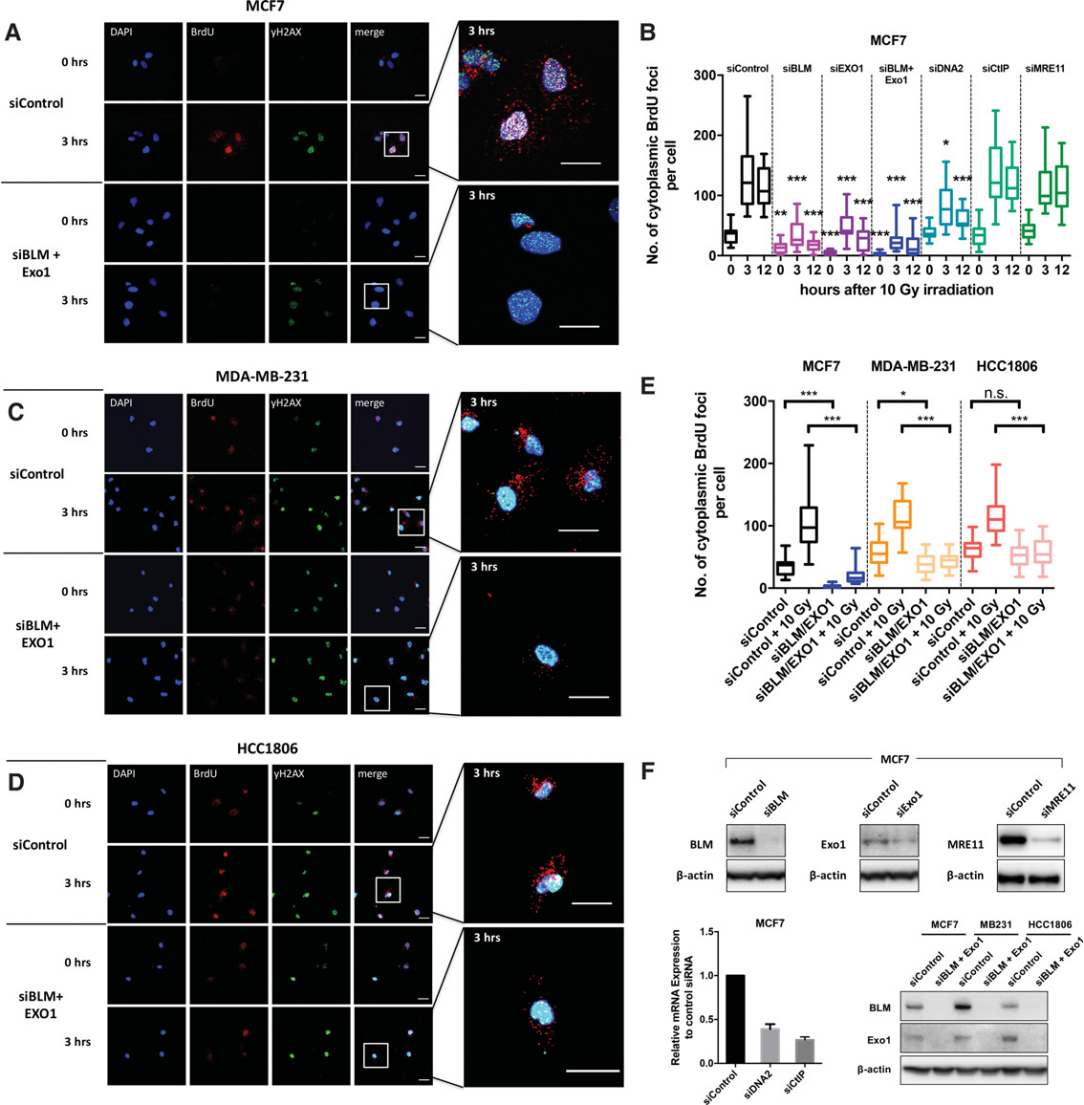
End resection factors are required for IR-induced accumulation of cytosolic ssDNA*.* (*A*) Representative confocal images of BrdU-incorporated MCF7 cells at the indicated times after treatment with 10 Gy of IR. Cells were pretreated with siRNA against EXO1 and BLM for a total of 48 h before irradiation. Cells were stained for DNA (DAPI; blue), BrdU (red), and yH2AX (green). Bars, 20 µm. (*B*) Quantification of BrdU foci outside the nucleus per cell in MCF7 cells treated with siRNA against the indicated DSB end processing and resection factors at the indicated time points following 10 Gy of IR. Boxes represent the upper and lower quartiles, the band represents the median, and the whiskers represent the minimum and maximum values in the data set. A minimum of 100 cells were scored per time point and grouped from three independent experiments. Statistical significance was determined by the Mann-Whitney *U*-test. Asterisks *above* boxes refer to the indicated time point versus the matching time point of siControl. (*C*,*D*) Representative confocal images of BrdU-incorporated MDA-MB-231 and HCC1806 cells at the indicated time points after 10 Gy of IR. Cells were pretreated with siRNA for a total of 48 h against BLM and EXO1 before irradiation. Cells were stained for DNA (DAPI; blue), BrdU (red), and yH2AX (green). Bars, 20 µm. (*E*) Box plot of BrdU focus quantification as in *B* but for MDA-MB-231 and HCC1806 cells treated with siRNA against BLM and EXO1 3 h after treatment with10 Gy of IR. (*F*) Western blot and quantitative RT–PCR analysis of the indicated cell lines to verify knockdown of the respective genes.

In an attempt to identify the source of the cytosolic ssDNA fragments, we investigated the effect of using siRNAs to deplete key factors involved in DNA DSB end processing and resection: BLM, EXO1, DNA2, CtIP, and MRE11. Depletion levels were >80% for BLM, >70% for EXO1, and ∼85% for MRE11 at the protein level and ∼70% for CtIP and ∼60% for DNA2 by quantitative RT–PCR analysis of mRNA levels (Fig. 2F).

Depletion of either BLM or EXO1 gave a substantial reduction in both nuclear and cytosolic ssDNA focus accumulation in both untreated and IR-treated MCF7 cells compared with the control siRNA. Codepletion of both factors and thus depletion of both major long-range resection pathways led to a further decrease and near-complete abrogation of the nuclear and cytosolic ssDNA staining seen at baseline and after IR treatment (Fig. 2A,B; Supplemental Figs. 5A, 6A,B). Moreover, on codepletion of Exo1 and BLM, an increase in nuclear γH2AX foci, both basal and IR-induced (24 h after irradiation), was observed, consistent with impairment of DSB repair (Supplemental Fig. 5B). Depletion of DNA2 also reduced cytosolic ssDNA accumulation, but this effect was less striking than EXO1 depletion. CtIP and MRE11 depletion had no significant effect (Supplemental Fig. 6A,B).

To confirm these results, we investigated whether the higher endogenous cytosolic ssDNA levels previously observed in TNBC cells (MDA-MB-231 and HCC1806 cell lines) (Fig. 1D) were also dependent on end resection factors. Depletion of BLM and EXO1 simultaneously resulted in a significant reduction of cytosolic DNA foci in the nonirradiated MDA-MB-231 samples and a highly significant reduction in the irradiated MDA-MB-231 and HCC1806 cell lines compared with the control siRNA-treated ones (Fig. 2C–E). These observations confirm the role of BLM and EXO1 in the generation of cytosolic DNA upon irradiation and imply a further mechanism of basal regulation.

We tested whether mitochondria (another potential source of release of cytosolic DNA fragments) were still intact after IR treatment using electron microscopy. No significant damage-related morphological changes were seen after treatment, except for dilated endoplasmic reticula (Supplemental Fig. 7A). Costaining of cells with MitoTracker and BrdU indicates that very few of the IR-induced ssDNA foci and mitochondria overlap (Supplemental Fig. 7B), suggesting that ssDNA mitochondrial replication intermediates do not explain the cytosolic ssDNA foci.

### The accumulation of cytosolic ssDNA after DNA damage and end resection activates an innate immune response

We determined the basal and IR-induced mRNA expression dynamics for a panel of type I IFN signaling target genes (*MX1*, *ISG15*, *OAS1*, *IFIT1*, *IFI6*, *IFI44*, and *BST2*) and the levels and phosphorylation status of the STAT1 protein. Higher expression levels of these genes are correlated with increased IRDS (Weichselbaum et al. 2008; Boelens et al. 2014). Phosphorylation of STAT1 (p-STAT1) at Tyr701 (Y701) is a well-established and sensitive activation marker of type I IFN signaling.

**Figure 3.**
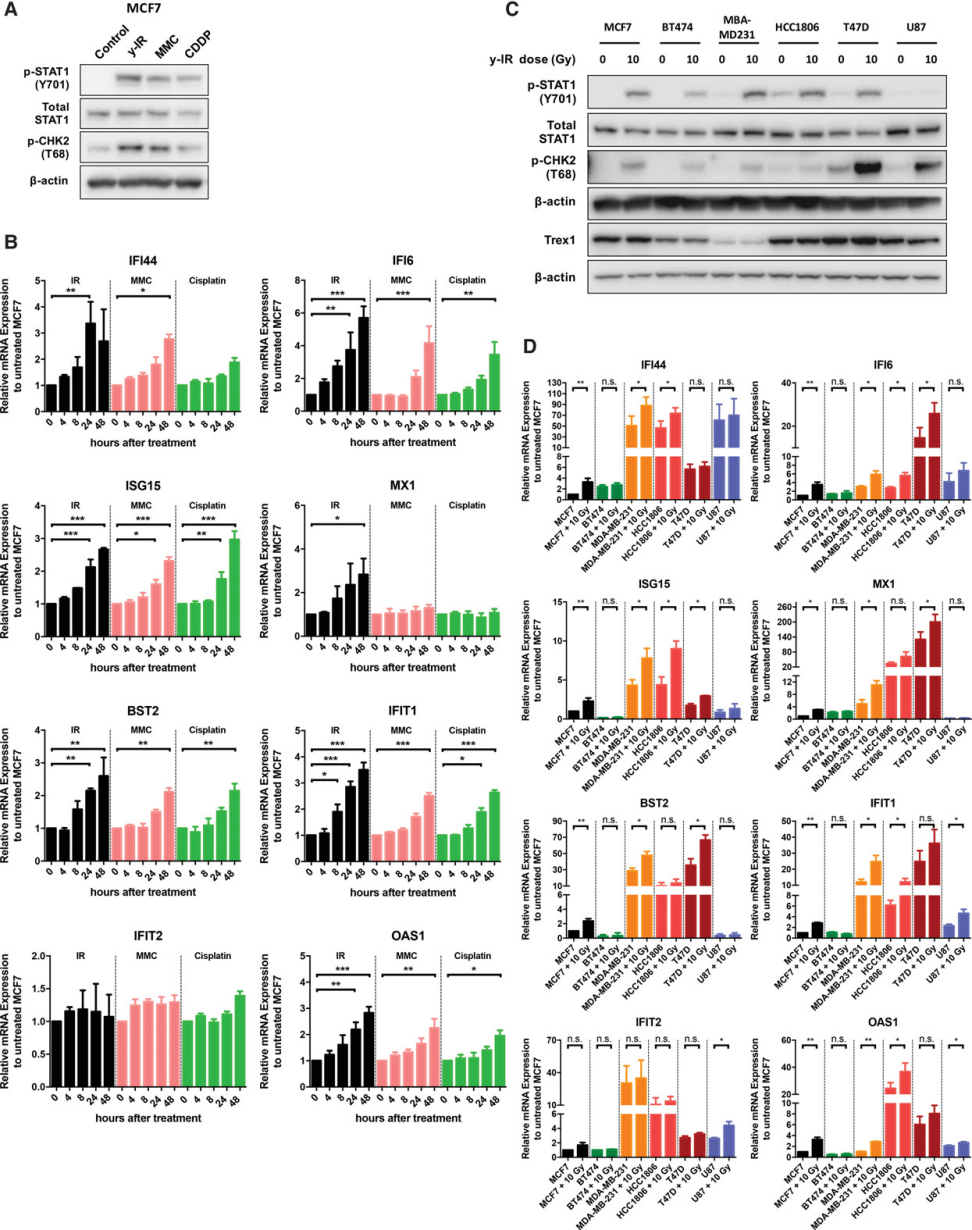
Accumulation of cytosolic ssDNA following DNA damage and end resection activates an innate immune response. (*A*) Western blotting of p-STAT1 (Y701), total STAT1, and p-CHK2 at T68 from MCF7 whole-cell extracts 24 h following 10 Gy of IR, 3 µM mitomycin C, and 15 µM cisplatin treatment. (*B*) Relative mRNA levels of the indicated ISGs over time in MCF7 cells following treatment with IR, 3 µM mitomycin C, and 15 µM cisplatin normalized to untreated MCF7. Bars represent mean values ± SD. *n* = 3 independent experiments. A two-tailed unpaired *t*-test was used to determine statistical significance. (*C*) Western blotting of p-STAT1 (Y701), total STAT1, p-CHK2 at T68, and Trex1 from the indicated cell lines 24 h after treatment with 10 Gy of IR. (*D*) Relative mRNA levels of the indicated ISGs 24 h after 10 Gy of IR in the indicated cell lines normalized to untreated MCF7. Bars represent mean values ± SD. *n* = 3 independent experiments. A two-tailed unpaired *t*-test was used to determine statistical significance.

IR, mitomycin C, and cisplatin treatment induced phosphorylation of STAT1 at Y701 within 24 h in MCF7 cells (Fig. 3A) as well as the expected induction of pCHK2(T68). Quantitative RT–PCR analysis confirmed activation of the panel of ISGs (Fig. 3B). With the exception of IFIT2, we observed a twofold to sixfold increase in ISG expression within 24 h of 10 Gy of IR in MCF7 cells. Significant induction could also be observed at the lower IR doses of 2 and 6 Gy (Supplemental Fig. 1E). Mitomycin C and cisplatin resulted in a delayed but similar fold change in mRNA expression except for MX1, which was selectively increased after IR treatment (Fig. 3B), consistent with the longer post-treatment periods required to induce cytosolic ssDNA.

Across the breast cancer cell line panel, including one radio-resistant glioblastoma cell line (U-87) used for comparison, the TNBC cell lines MDA-MB-231 and HCC1806 had the highest basal and post-IR treatment levels of p-STAT1 protein (Fig. 3C). Relative mRNA expression among the cell lines showed further heterogeneity in ISG expression (Fig. 3D). All of the examined ISGs showed higher endogenous and IR-induced expression levels in at least one of the TNBC cell lines (MDA-MB-231 and HCC1806) compared with MCF7 (Fig. 3D). Interestingly, MDA-MB-231 cells exhibit lower levels of Trex1 (see below for the importance of Trex1 in cytosolic ssDNA response), where these results and the higher levels of pSTAT1 positively correlated with the more abundant cytosolic ssDNA foci seen in these cells (Fig. 1D).

**Figure 4.**
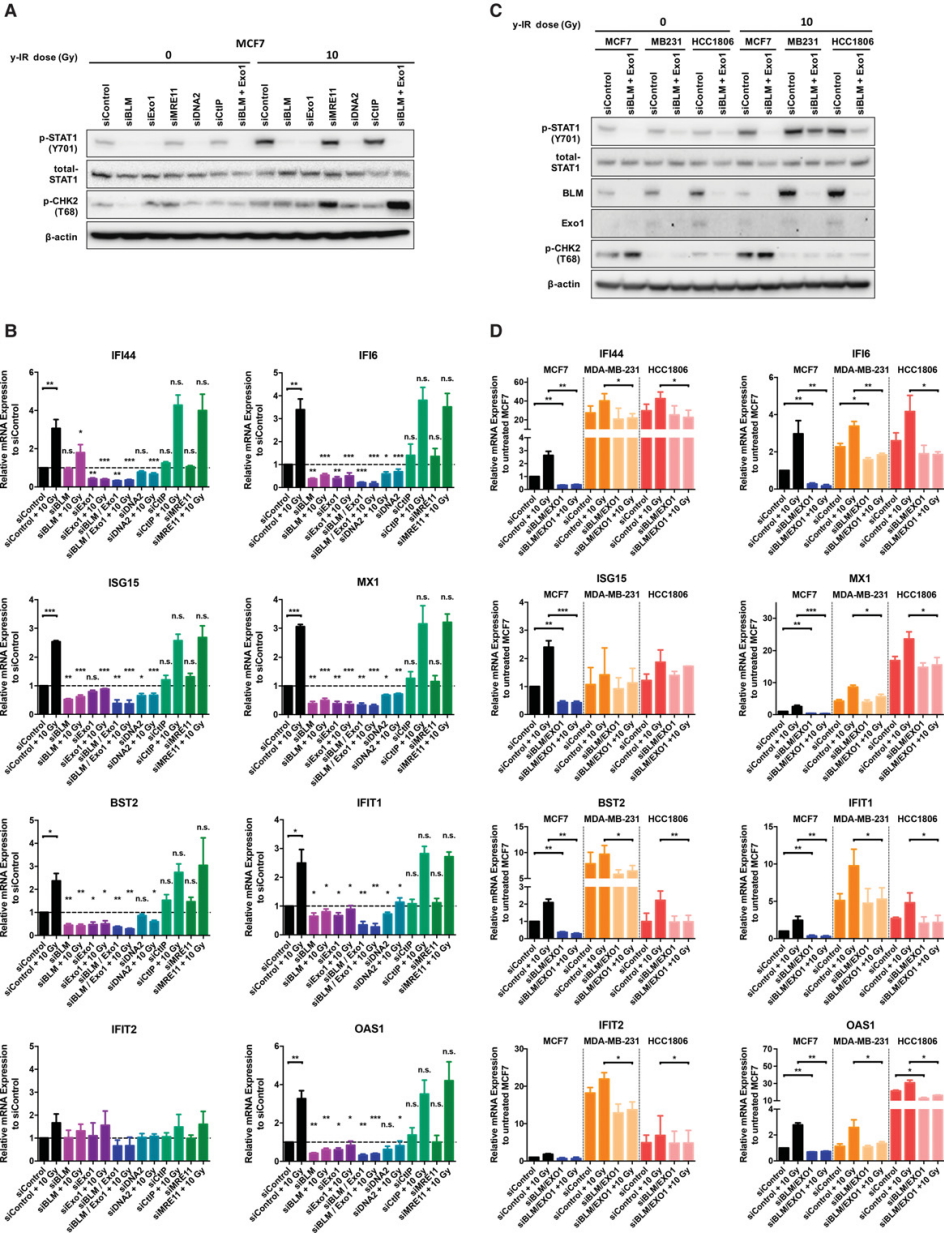
Accumulation of cytosolic ssDNA after DNA damage and end resection activates an innate immune response. (*A*) Western blotting of p-STAT1 at Y701, total STAT1, and p-CHK2 at T68 from MCF7 cells treated with siRNA against the indicated DSB end processing and resection factors and 24 h after treatment with 10 Gy of IR. (*B*) Relative mRNA levels of the indicated ISGs in MCF7 cells treated with siRNA against the indicated DSB end processing and resection factors and 24 h after treatment with 10 Gy of IR. Bars represent mean values ± SD. *n* = 3 independent experiments. Two-tailed unpaired *t*-test was used to determine statistical significance. Asterisks *above* columns refer to respective siRNA (untreated) versus siControl (untreated) and respective siRNA (10 Gy treated) versus siControl (10 Gy treated). (*C*) Western blotting of p-STAT1 at Y701, total STAT1, BLM, EXO1, and p-CHK2 at T68 from the indicated cell lines and 24 h after treatment with 10 Gy of IR. (*D*) Relative mRNA levels of the indicated ISGs following depletion of BLM and EXO1 and 24 h following 10 Gy of IR in the indicated cell lines normalized to untreated MCF7. Bars represent mean values ± SD. *n* = 3 independent experiments. A two-tailed unpaired *t*-test was used to determine statistical significance.

Single BLM, EXO1, and DNA2 depletion reduced pSTAT1 protein levels prior to and after IR, while depletion of CtIP and MRE11 showed no effect (Fig. 4A); corresponding decreases were also observed for ISG mRNA expression levels. Double depletion of BLM and EXO1 significantly reduced endogenous and post-IR treatment ISG levels to between ∼38% (ISG15) and ∼21% (IFI6) depending on the ISG, with the exception of IFIT2 (Fig. 4B). Double depletion of BLM and EXO1 also led to a reduction of basal and post-IR treatment STAT1 phosphorylation at Y701 (Fig. 4C) and ISG expression levels in the TNBC cell lines, although this effect was less dramatic than in MCF7 cells (for MDA-MB-231, down to between ∼66% [BST2] and ∼52% [OAS1]; for HCC1806, down to between ∼67% [IFIT2] and ∼43% [BST2]) (Fig. 4D). We conclude that long-range DSB end resection plays a key role following irradiation in generating the cytosolic ssDNA fragments that activate STAT1 and then the ISGs associated with IRDS.

### IR-induced cytosolic DNA fragments are ∼20 nucleotides (nt) in length

**Figure 5.**
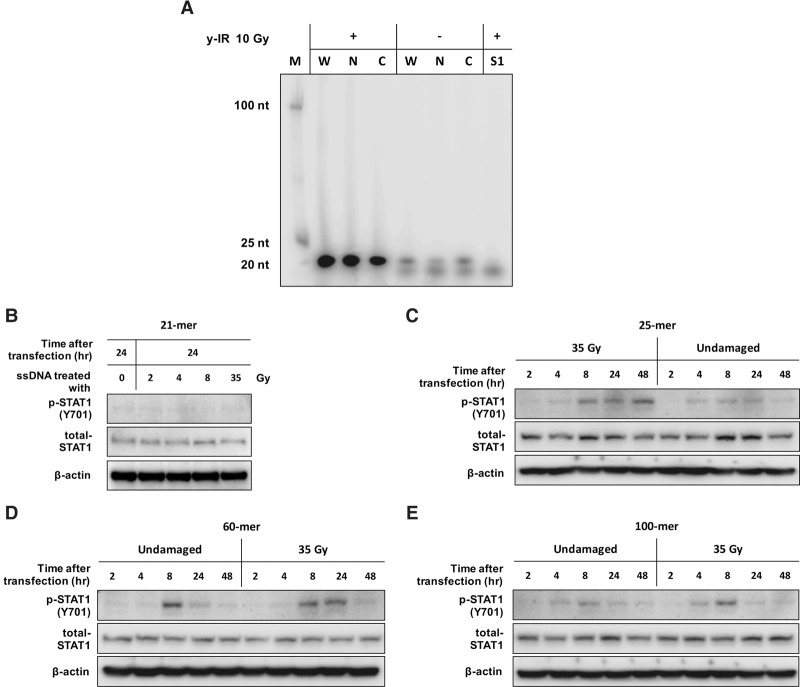
Defining the minimum DNA fragment size required to activate an IFN response and determining the size of IR-induced cytosolic DNA fragments. (*A*) Purified DNA samples obtained from irradiated and nonirradiated MCF7 whole-cell lysate (W) and nuclear (N) and cytosolic (C) fractions labeled on the 5′ end with γ-^32^P-dATP. All samples were treated with RNase A, and the S1 lane was additionally treated with S1 nuclease prior to labeling before analysis on a 6% nondenaturing polyacrylamide gel. (*B*) Western blotting of p-STAT1 (Y701) and total STAT1 for MCF7 whole-cell extracts 24 h after transfection with irradiated and nonirradiated 21-mer. (*C*–*E*) Western blotting of p-STAT1 at Y701 and total STAT1 for whole-cell extract prepared from MCF7 cells transfected with IR-damaged and undamaged 25-mer, 60-mer, and 100-mer oligonucleotides.

Using subcellular fractionation and immunocapture techniques, we found that the average size of cytosolic ssDNA is ∼20 nt (Fig. 5A). To determine the characteristics needed for ssDNA to engage this pathway, we transfected MCF7 cells with IR-treated and untreated ssDNA oligonucleotides ranging from 21-mer to 100-mer. While 21-mer transfected cells did not induce appreciable STAT1 phosphorylation (Fig. 5B), larger sequence-related oligonucleotide ssDNA fragments lead to increased phosphorylation levels (Fig. 5C–E), suggesting that a minimum length of ssDNA is needed to elicit downstream type I IFN signaling. Interestingly, although the minimum size required to induce STAT1 phosphorylation was 25 nt, transfection of higher-molecular-weight substrates, including 60-mer and 100-mer, did not further augment this response. Furthermore, in vitro irradiation of fragments before adding them to cells showed increased potency in phosphorylation of STAT1 (Fig. 5C–E), indicating that the IR-induced oxidative/chemical modification in resected fragments might augment their ability to stimulate ISGs. However, a caveat here is that the inability of transfected 21-mer ssDNA to elicit IFN signaling raises the possibility that the presentation of DNA fragments generated during biological processing might differ markedly from that of those artificially introduced into cells, possibly by virtue of other factors associating with DNA fragments escaping the nucleus.

### Trex1 degrades IR-induced cytosolic ssDNA fragments

**Figure 6.**
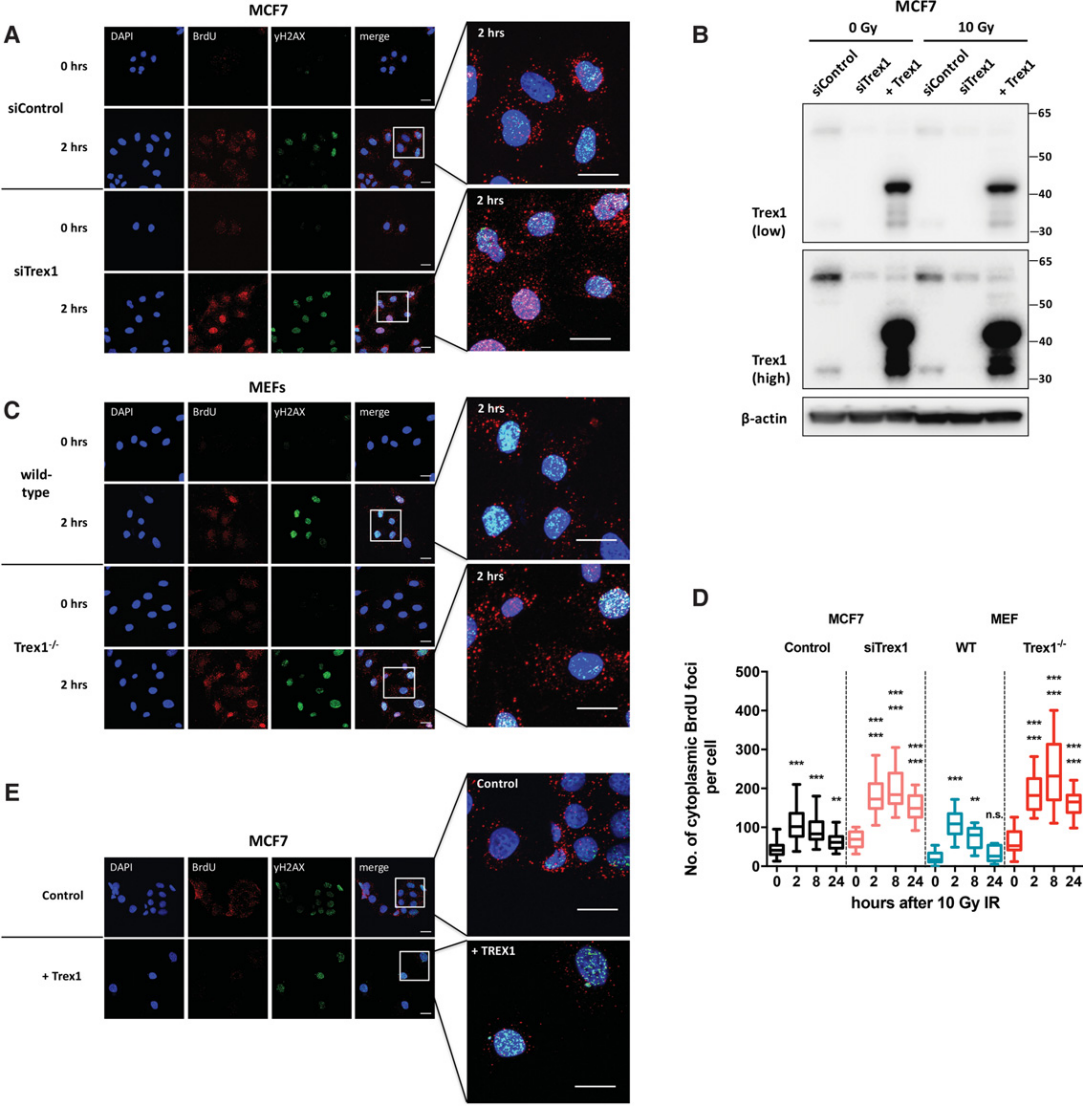
Trex1 degrades IR-induced cytosolic ssDNA. (*A*) Representative confocal images of BrdU-incorporated MCF7 cells at the indicated time points. Cells were treated with siRNA against Trex1 for 48 h before being irradiated with 10 Gy of IR and were subsequently stained for DNA (DAPI; blue), BrdU (red), and yH2AX (green). Bars, 20 µm. (*B*) Western blot analysis of MCF7 to verify depletion and overexpression of Trex1. Images are shown at low and high exposure. (*C*) Representative confocal images of BrdU-incorporated wild-type and *Trex1*^−/−^ MEF cells at the indicated time points. Cells were stained for DNA (DAPI; blue), BrdU (red), and yH2AX (green). Bars, 20 µm. (*D*) Quantification of nonnuclear BrdU foci per cell in MCF7 treated with siRNA against Trex1 and wild-type and *Trex1*^−/−^ MEF cells at the indicated time points following 10 Gy of IR. Boxes represent the upper and lower quartiles, the band represents the median, and the whiskers represent the minimum and maximum values in the data set. A minimum of 100 cells were scored per time point and grouped from three independent experiments. Statistical significance was determined by the Mann-Whitney *U*-test. Asterisks *above* each box refer to the indicated time point after 10 Gy versus the 0-h control of the same cell line (*bottom*) and the indicated time point after 10 Gy treatment versus the same time point in the parental cell line (*top*). (*E*) Representative confocal images of BrdU-incorporated Trex1-overexpressing MCF7 cells after 10 Gy of IR. Cells were stained for DNA (DAPI; blue), BrdU (red), and yH2AX (green). Bars, 20 µm.

Trex1 degrades endogenous cytosolic DNA fragments and has a substrate preference for ssDNA and dsDNA containing mispaired 3′ termini (Yang et al. 2007). It has been suggested that in Trex1-deficient cells, the accumulated basal ssDNA fragments are derived from endogenous retroelements or aberrant reverse transcription intermediates (Stetson et al. 2008). We investigated whether IR-induced ssDNA fragments are Trex1 substrates through siRNA-mediated depletion of Trex1 in MCF7 cells (Fig. 6A,B). Trex1-depleted cells showed increased cytosolic ssDNA accumulation in both irradiated and unirradiated samples (Fig. 6A,D). To verify these findings, we also assessed the effects of IR treatment of wild-type and *Trex1*^−/−^ mouse embryonic fibroblasts (MEFs). Similar to the results obtained in human cells, Trex1 deficiency in MEFs resulted in enhanced accumulation of IR-induced ssDNA. *Trex1*^−/−^ MEFs had significantly higher basal and post-treatment cytosolic ssDNA foci compared with the wild-type cells (Fig. 6C,D). Furthermore, overexpression of Trex1 in MCF7 cells led to an opposing effect, strikingly reducing cytosolic DNA foci (Fig. 6E) but not nuclear foci. We conclude that Trex1 represents a major cytosolic activity required to degrade the damage-released ssDNA fragments.

### Role of Trex1 and cGAS–STING in the induction of the IFN-related DNA damage signature and sensitivity to IR

**Figure 7.**
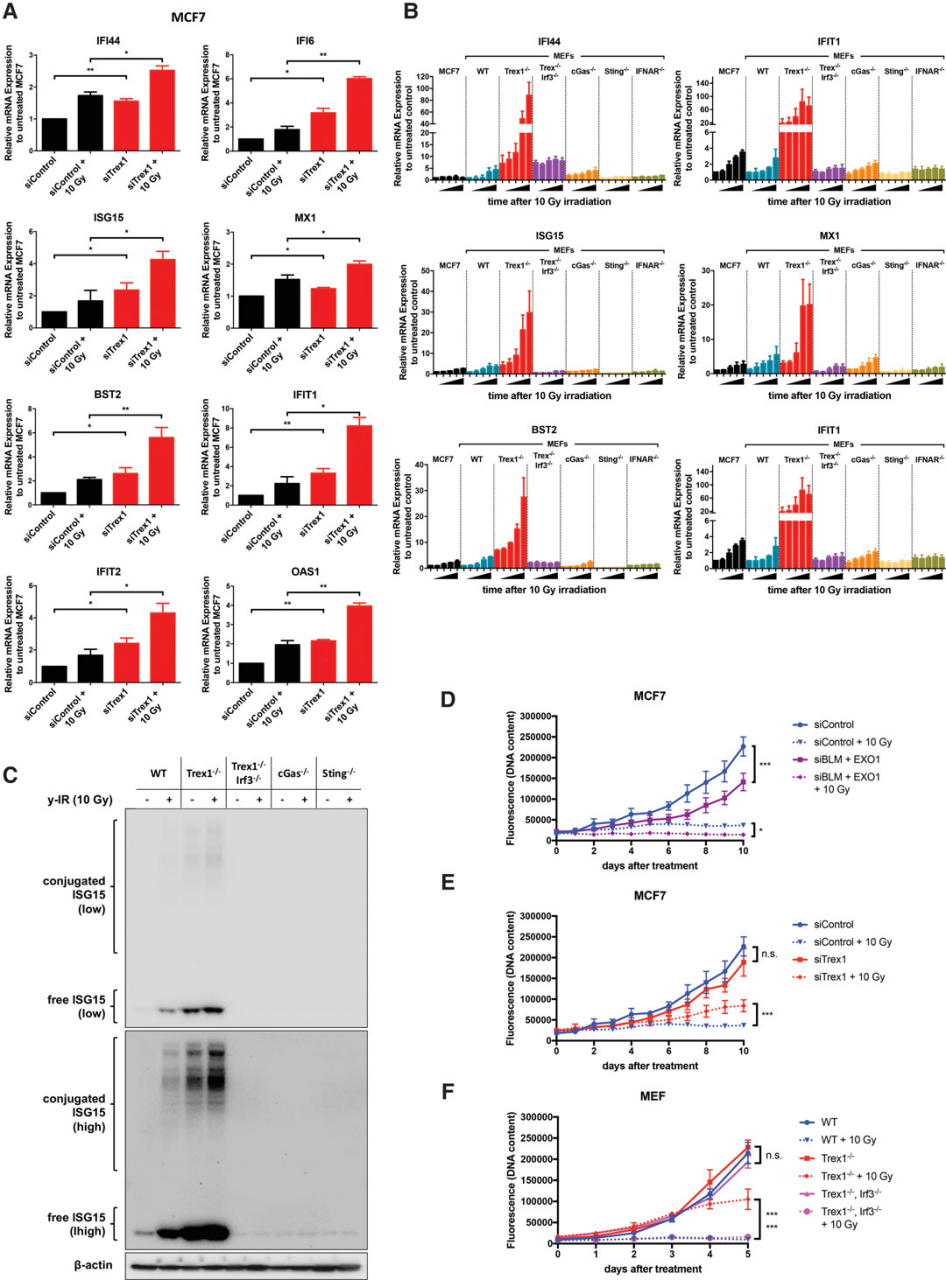
Role of Trex1 and the cGAS–STING pathway on IFN-related DNA damage signature and sensitivity to IR. (*A*) Relative mRNA levels of the indicated ISGs after depletion of Trex1 and 24 h following 10 Gy of IR in MCF7 cells normalized to siControl-treated MCF7. Error bars represent mean values ± SD. *n* = 3 independent experiments. A two-tailed unpaired *t*-test was used to determine statistical significance. (*B*) Relative mRNA levels of the indicated ISGs over time in MCF7, wild type, *Trex1*^−/−^, *Trex1*^−/−^ *Irf3*^−/−^ double knockout, *cGas*^−/−^, *Sting*^−/−^, and *Ifnar*^−/−^ after 10 Gy of IR. Treated MCF7 was normalized to untreated MCF7, while MEFs were normalized to untreated wild type. Bars represent mean values ± SD. *n* = 3 independent experiments. (*C*) Western blotting of ISG15 from whole-cell extracts prepared from the indicated cell lines 24 h after 10 Gy of IR. Images are shown at low and high exposure. (*D*) Growth curves of MCF7 cells depleted for BLM and EXO1 after 10 Gy of IR. PicoGreen was used to determine DNA content. Statistical significance between the data sets was determined using a two-way ANOVA test. (*E*) Growth curves of MCF7 cells depleted for Trex1 after 10 Gy of IR. (*F*) Growth curves of wild-type, *Trex1*^−/−^, Trex1, and Irf3 double-knockout (DKO) MEF cells after treatment with 10 Gy of IR. Asterisks at the *right* of the growth curves refer to 10-Gy-treated *Trex1*^−/−^ versus 10-Gy-treated wild-type (*top*) and 10-Gy-treated *Trex1*^−/−^ versus 10-Gy-treated double knockout (*bottom*).

In accordance with the ability of Trex1 to degrade cytosolic ssDNA, its depletion in MCF7 cells greatly increased IR-induced ISG mRNA expression levels (Fig. 7A). *Trex1*^−/−^ MEF cells also showed a striking increase in ISG expression after IR compared with wild-type cells (Fig. 7B). However, *Trex1* and *Irf3* double-knockout MEF cells showed no increase in ISG expression levels after IR treatment compared with *Trex1*^−/−^ single-knockout cells (Fig. 7B), demonstrating that DNA damage-induced Trex1-mediated ISG expression is driven by IRF3. Although higher basal mRNA levels for IFI44, IFIT2, and BST2 were seen compared with the wild-type cells (approximately fourfold for IFI44 and approximately eightfold for IFIT2 and BST2) in the double mutant, they were still lower compared with *Trex1*^−/−^ cells (Fig. 7B). *cGas*^−/−^ cells show dampened mRNA expression levels after IR compared with wild-type cells, while *Sting*^−/−^ cells exhibited complete loss of IR-induced ISG expression (Fig. 7B). To further validate the quantitative RT–PCR results, protein levels of ISG15 prior to and after IR treatment were assessed for all MEF cells. An increase in free ISG15 was observed after IR treatment in both wild-type and *Trex1*^−/−^ cells, while the latter showed higher basal protein levels compared with wild type. *Trex1* and *Irf3* double-knockout, *cGas*^−/−^, and *Sting*^−/−^ cells did not show any changes in ISG15 protein levels after treatment (Fig. 7C), although all of them showed cytosolic ssDNA accumulation (Supplemental Fig. 8). In order to definitively examine whether type I IFN signaling underlies the observed effects, we examined the effects of IR treatment of MEFs disrupted for the type I IFN receptor (*Ifnar*^−/−^ cells). While these cells accumulate equivalent levels of cytosolic ssDNA following irradiation (Supplemental Fig. 8A,B), they almost completely failed to induce expression of ISGs. Together, these data demonstrate that the cytosolic ssDNA accumulating as a consequence of DSB resection activates a canonical IFN-mediated antiviral-like response and that it is a substrate for Trex1 degradation. Finally, we examined the effect of pharmacological inhibition of TBK1 (by BX795), a master regulator of IRF3 activation (Clark et al. 2009) that dramatically reduced the induction of IFN-inducible genes (Supplemental Fig. 8C).

To examine the effects of the cytosolic DNA accumulation on cell viability after IR treatment, a PicoGreen cell proliferation assay was performed. Treatment with siRNA to either BLM and EXO1 in MCF7 cells led to slower growth and some sensitization to 6 and 10 Gy of IR (Supplemental Fig. 9D), and codepletion of BLM and EXO1 and therefore loss of long-range resection further reduced survival at both 6 and 10 Gy (Fig. 7D; Supplemental Fig. 9C,D). This result reflects the contribution of a DNA repair defect in conjunction with viability after IR along with any contribution of the IFN response to cell viability. In order to dissect the specific impact of cytosolic DNA processing and signaling, MCF7 cells depleted for Trex1 as well as MEFs harboring Trex1 disruption were studied. Both showed a significant resistance to treatment with 10 Gy of IR, indicating that response to the accumulation of cytosolic DNA might enhance cell survival following such damage (Fig. 7E). We next examined the consequences of eliminating ISG signaling in Trex1 wild-type and disrupted cells through the comparison of the sensitivity of Trex1^−/−^ cells with those also disrupted for Irf3. Following IR treatment, only *Trex1*^−/−^ MEFs continued to grow, and the double knockout of *Trex1* and *Irf3* (*Trex1*^−/−^ *Irf3*^−/−^ cells) resensitized the MEFs to IR (Fig. 7F). TNBC cell lines also showed similar results, except that Trex1 knockdown in HCC1806 did not mediate better survival (Supplemental Fig. 9). Notably, this cell line already had the highest basal levels of ssDNA foci.

### The relationship of Trex1 and DSB resection to survival probability in breast cancer patients

**Figure 8.**
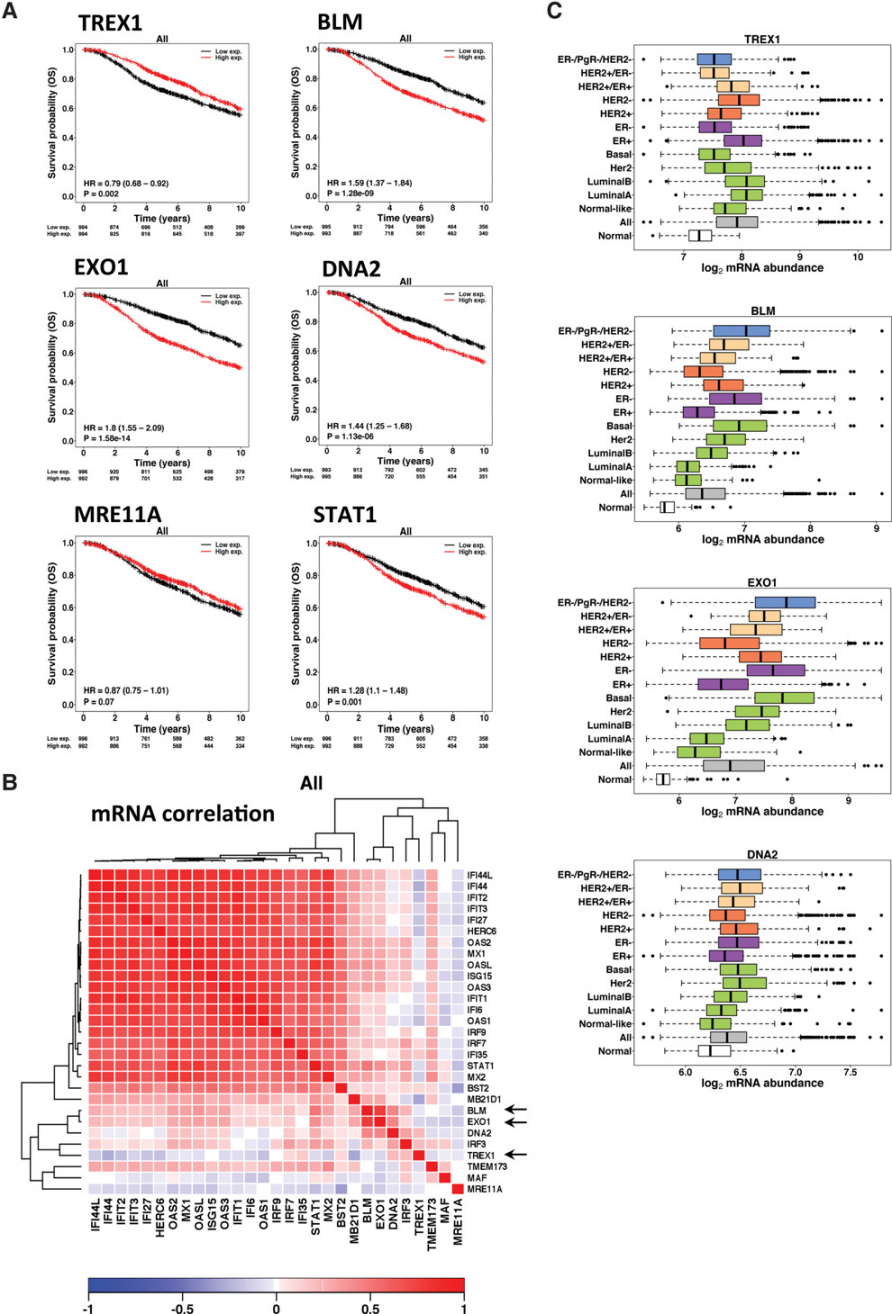
Role of Trex1 and resection factors on survival probability in breast cancer patients. (*A*) The Metabric breast cancer data set was used to determine whether the expression of candidate gene products identified in this study show association with clinical outcome (10-yr overall survival). Kaplan-Meier survival curves and Cox proportional hazards model statistics are shown for Trex1, BLM, EXO1, DNA2, Mre11, and STAT1. For each gene, the breast cancers were classified into high- or low-expressing groups based on median dichotomisation on mRNA abundance levels. (*B*) The set of IRDS genes induced by radiation therapy was analyzed in the Metabric data set to investigate correlations between the gene expression patterns. The heat map shows mRNA abundance correlation/coexpression (Spearman's rho) of prominent IRDS genes and DNA resection factors. (Red) Positive correlation; (blue) negative correlation. The arrows in the heat map indicate the strong association of EXO1 and BLM expression with the expression of IFN response genes, the inverse correlation of Trex1 expression, and the lack of any effect of MRE11A. In addition, the set of IRDS genes is clearly highly correlated with each other. (*C*) mRNA abundance across different breast cancer subtypes for Trex1, BLM, EXO1, and DNA2. (All) mRNA abundance across all breast cancer patients; (Her2) PAM50 subtype; (HER2^+/−^) immunohistochemistry-based status; (ER^+/−^) immunohistochemistry-based groups. PgR^+/−^ groups were derived using mRNA abundance data.

Analysis of Metabric breast cancer data (Curtis et al. 2012) showed that high Trex1 mRNA was associated with good prognosis. Conversely, high levels of BLM or EXO1 transcripts were associated with poor prognosis, EXO1 having a stronger effect than BLM. High levels of DNA2 also showed an association with poor outcome but less so than BLM or EXO1, recapitulating the observations in the cell lines. Similarly, MRE11 showed no statistically significant association with outcome. Finally, STAT1 RNA levels showed a significant correlation, where high levels were correlated with poor outcome, notwithstanding the fact that STAT1 is clearly also regulated at the post-transcriptional level. This may represent the association of mRNA with basal levels of protein (Fig. 8A).

We next investigated whether the RNAs expressed for a panel of IRDS genes, including those analyzed in our cell lines, correlated with each other. It was clear that there is a strong pattern of coexpression for this response pathway. Additionally, we found that high expression of BLM and EXO1 was also correlated with high expression of IRDS-associated genes, as was high IRF3 and DNA2. There was no relationship with MRE11, and Trex1 was inversely related to these sets of genes (Fig. 8B).

Assessing the individual subtypes of breast cancer, it is clear that EXO1 and BLM were, in general, much more highly expressed than in normal breast tissue, whereas Trex1 expression was usually in a similar range. However, in each case, the triple-receptor-negative, basal, ER-negative, and HER2-positive phenotypes and luminal B had higher levels of expression of BLM and EXO1. Conversely, Trex1 levels were higher in ER-positive breast cancers and HER2-negative breast cancers and were associated with a better prognosis (Fig. 8C; Supplemental Table 1).

Poly-ADP-ribose polymerases (PARPs; principally PARP1–3) play an important role in the repair of DNA single-strand breaks, base damage, and also DSB repair and fork repair and restart (Caldecott 2014). Moreover, Trex1 interacts with PARP1. Therefore, we examined whether the recently clinically approved PARP inhibitor (selective for PARP1 and PARP2) olaparib (Lynparza) had an effect on the accumulation of cytoplasmic ssDNA after IR and the consequences for induction of the IFN response and cell survival. Olaparib treatment increased both basal and IR-induced levels of cytosolic ssDNA (Supplemental Fig. 10A,B), consistent with its interference with DNA repair processes. A small but not statistically significant increase in the basal induction of the panel of ISGs could be observed (Supplemental Fig. 10C). However, a substantial and significant increase in the induction of the same gene panel could be observed following IR treatment in the presence of olaparib compared with irradiation in its absence. This was associated with reduced cell survival following 6 Gy of IR, suggesting that previously reported interactions between PARP inhibitors and radiation sensitivity in tumor models (Veuger et al. 2003; Loser et al. 2010) potentially result from a complex interplay between altered DNA repair dynamics and increased engagement of the IFN pathway, which in turn might be modulated by PARP inhibition.

## Discussion

Here we show that, in breast cancer cells, cytosolic ssDNA is present at various endogenous basal levels but can be dramatically increased by therapeutically relevant DNA-damaging agents, in particular IR. Our results indicate that DSB end resection activities contribute to the generation of ssDNA fragments that accumulate in the cytosol following exposure to DSB-inducing DNA damage. Both basal and IR-induced cytosolic DNA fragment levels are dramatically reduced when both major long-range resection pathways are lost in BLM- and EXO1-depleted cells.

Thus, the response to DNA DSB damage may proceed via two interrelated mechanisms. The first is due to defects in DSB resection. To our knowledge, the requirement for BLM and EXO1 in cells for end resection after the induction of frank DSBs following IR has not been demonstrated previously. No dramatic reduction in the generation of cytosolic ssDNA was observed in Mre11-depleted cells. Mre11 is known to play an important role in resection (Cejka 2015), but its requirement in resection of IR-induced DSBs has been reported recently to be only partial in genomically disrupted human cells (Hoa et al. 2015).

The second mechanism is through the engagement of the IFN I antiviral response pathway, as identified here. We used cell lines that were unable to degrade the cytosolic DNA (Trex1-deficient cells). Strikingly, the accumulation and persistence of cytosolic DNA in Trex1-deficient MCF7 breast cancer cells or *Trex1*^−/−^ MEFs led to an increase in radio resistance. This suggests that the ISG signaling induced by the accumulated ssDNA fragments promotes survival following IR. To examine whether the effect is directly mediated through ISGs and the type I IFN response, we tested *Trex1*^−/−^ MEFs that are also disrupted for Irf3 and therefore are not able to invoke the IFN transcription program. Loss of Irf3 in *Trex1*^−/−^ cells completely reversed the radio resistance observed in the Trex1-deficient background, the role of the IR-protective and proliferation-enhancing IFN response.

Our study contributes to accumulating literature linking DNA damage processing to engagement of cytosolic DNA-sensing pathways. Indeed, ATM deficiency and the resulting unrepaired DNA damage result in the accumulation of cytosolic DNA and STING-dependent IFN induction (Hartlova et al. 2015). Similarly, treatment of mouse B-cell lymphoma cells and human lung carcinoma cells with a replication inhibitor showed induction of cytosolic DNA (Shen et al. 2015). Further findings suggest the possibility that release of MUS81-induced cytosolic DNA by prostate cancer cells contributes to STING activation (Ho et al. 2016), where such fragments might be a by-product of MUS81-mediated replication fork processing in these cells (Ciccia et al. 2008). Moreover, RNase H2 deficiency, the most frequent cause of the autoinflammatory disease Aicardi-Goutières syndrome (AGS), triggers cGAS/STING activation (Crow and Manel 2015; Mackenzie et al. 2016; Pokatayev et al. 2016). It is possible that dsDNA fragments accumulate in this setting as a by-product of repair reactions associated with strand breaks that could be triggered by conflicts between DNA replication and transcription due to the accumulation of genomic RNA–DNA hybrids (R loops) in RNase H2-deficient cells or to defective ribonucleic acid excision repair leading to persistent genomic DNA strand breaks (Mackenzie et al. 2016). These findings are in line with our present discoveries that implicate end resection during HR in the release of the ssDNA that activates cGAS and STING (Pepin et al. 2016), although some residual ISG activation was observed in cGas-deficient cells, raising the possibility that additional cytoplasmic factors are involved in initial ssDNA sensing.

While dsDNA is the most potent activator of the cGAS–STING pathway (Sun et al. 2013), through its tight interaction with cGAS, ssDNA is capable of binding cGAS with a K_d_ ∼10-fold higher than that of dsDNA (Kranzusch et al. 2013). In this regard, it is notable that in vitro irradiation of the transfected oligonucleotides increased their potency to elicit a response, suggesting that the presence of IR-induced chemical and oxidative damage might either enhance the nonself recognition pattern of the DNA fragments by cGAS or render them refractory to digestion by Trex1, increasing their half-life, and, indeed, a recent report indicates that DNA containing oxidative lesions is resistant to Trex1 digestion (Gehrke et al. 2013). Moreover, another link was made recently between Trex1 and DNA repair factors, since it was reported that RPA and Rad51, two ssDNA-binding proteins critical for DSB repair, counteract the export of ssDNA generated in the nucleus as part of a cell-intrinsic mechanism to prevent activation of the IFN response (Wolf et al. 2016). It appears likely that, under conditions of no induced genomic stress, these factors are sufficient to sequester ssDNA fragments arising as by-products of damage processing, but, when significant levels of exogenous damage are induced, as in our current report and in the case of cancer treatment, this system is saturated, leading to the leakage of ssDNA into the cytosol. It will now be of considerable interest to define the origin of the ssDNA fragments at the sequence level. This will be a challenging area for further investigation, since our current enrichment strategies produce an extremely low yield of cytosolic ssDNA fragments, as other investigators have reported recently (Mackenzie et al. 2016).

Our work provides a potential explanation for the inferences made regarding the importance of IRDS in determining the DNA damage response in cancer cell lines across numerous tumor types and clinically also in cancer patients. There, previous studies suggest that the presence of an IRDS correlates with resistance to therapeutic DNA damage and also to reduced long-term survival in breast cancer patients (Weichselbaum et al. 2008). Moreover, high levels of RNA polymerase θ (Pol θ), a protein important for DSB repair by alternative end-joining (altEJ), are associated with poor outcome in breast, ovarian, and other tumors, and recent analysis demonstrates that EXO1 and BLM1 cocluster with Pol θ within a cohort of genes strongly correlated with poor outcome in invasive breast carcinomas (Wood and Doublie 2016).

Our work is consistent with the correlative data reported in breast cancer, linking high expression of the IRDS genes to clinical chemoresistance and radio resistance. However, this also shows that high induction to similar levels can occur in cells with low baseline levels, with important implications for early monitoring of response in clinical studies selecting inhibitors of this pathway. Our analysis of clinical data available from the Metabric cohort (Curtis et al. 2012) suggests that profiles before therapy could be relevant for selecting patients for drugs to modify their IFR or antibodies against the IFNα and IFNβ receptors. However, this approach might be more fruitful if analysis was undertaken both before and after the first dose of radiation therapy, as our data show that some cell lines have high basal levels of type I IFN signaling target genes, as do some patients, and this is associated with increased resistance, whereas other cell lines have low basal levels but can be induced to higher levels. Nevertheless, the final fold induction might be the critical indicator and biomarker of the effects on outcome, and so early dynamic monitoring of response should be considered to evaluate the role of this in prospective studies. This has important implications for the design of new interventions to blockade the DNA-induced IRDS pathway. Our observations may explain the poor previous results of combinations of IFNs—introduced in the 1990s—with several forms of chemotherapy and radiotherapy in solid tumors (besides melanoma and renal cancer) for which no adequate explanation was provided, as extensively reviewed by Santhanam et al. (2002).

Finally, our study makes a strong argument for the continued development of BLM inhibitors and the initiation of efforts to inhibit EXO1 (Nguyen et al. 2013). Such inhibitors could attenuate the repair of IR-induced DSBs, which in itself might improve a therapeutic index of radiotherapy and DNA-damaging chemotherapeutics, but, in addition, such agents may have a key role in attenuating the IRDS, further enhancing therapeutic response.

## Materials and methods

### Cell lines and procedures

Cancer cell lines (MCF7, BT-474, MDA-MB-231, HCC1806, T-47D, and U-87) were obtained from American Type Culture Collection. MEFs were obtained from embryonic day 12.5 (E12.5)–E14.5 embryos as described before (Bridgeman et al. 2015). *Trex1*^−/−^ MEFs were provided by T. Lindahl (Yang et al. 2007). *cGas*^−/−^ mice were described before (Bridgeman et al. 2015), *Sting*^−/−^ mice were provided by J. Cambier, and *Ifnar*^−/−^ mice were from C. Reis e Sousa (all on a C57BL/6 background) (Jin et al. 2011). *Trex1*^−/−^; *Irf3*^−/−^ MEFs were a kind gift from D. Stetson (C57BL/6 background) (Stetson et al. 2008). All cell lines were cultured in DMEM containing 10% fetal bovine serum (FBS) at 37°C and 5% CO_2_. Cells were irradiated with a 137Cs irradiator (IBL 637, CIS Bio International); irradiation was at a dose of 10 Gy unless otherwise specified. For BrdU labeling, cells were labeled with 10 mM BrdU (BD Bioscience) for 1.5 cell cycles (determined individually for each cell line) (see Supplemental Table 5) before being washed with PBS and returned to BrdU-free medium. For olaparib (Lynparza) and BX795 (Sigma) treatment, cells were incubated with 2.5 µM olaparib for 16 h or 0.5 µM BX795 for 2 h before being washed with PBS and returned to fresh medium.

### Fluorescence microscopy and cell imaging

Cells grown on glass coverslips were permeabilized for 2 min with CSK buffer (10 mM PIPES at pH 6.8, 300 mM sucrose, 100 mM NaCl, 1.5 mM MgCl2, 0.5% Triton) on ice and fixed with 4% paraformaldehyde in PBS for 10 min at room temperature. MitoTracker (Thermo Fisher) staining was performed at 200 nM for 20 min at 37°C according to the manufacturer's instructions. Where indicated, samples were pretreated for 30 min at 37°C with 200 mg/mL RNase A (Qiagen) and with S1 nuclease (Promega) or P1 nuclease (Sigma) plus reaction buffer. Samples were blocked for 30 min with 10% FBS in PBS and incubated with the indicated primary antibodies (0.1% FBS in PBS). Unbound primary antibodies were removed by washing four times for 5 min each in PBS followed by incubation with secondary antibodies for 45 min at room temperature. Slides were then washed four times for 5 min each in PBS before mounting with VectaShield mounting medium (Vector Laboratories) containing DAPI. Images were taken using a 63× objective on a Zeiss LSM 510 laser-scanning confocal microscope. Image analysis was carried out with Fiji (ImageJ) software.

### Cell extracts and immunoblotting

To prepare whole-cell extracts, cells were lysed in ice-cold lysis buffer (150 mM sodium chloride, 20 mM Tris at pH 7.5, 1% Triton X-100, 0.5% sodium deoxycholate, 0.1% SDS [sodium dodecyl sulfate], and 1 mM EDTA) plus protease inhibitor cocktail and phosphatase inhibitor cocktail (Roche). Protein concentration of lysates was measured by Bradford assay. Cell lysates were separated by 4%–12% Bis-Tris SDS-PAGE (Thermo Fisher), blotted onto polyvinylidene difluoride membrane (GE healthcare), blocked in TBST–10% dried skimmed milk, and incubated with antibodies in TBST–5% dried skimmed milk. Signals were detected by horseradish peroxidase-conjugated secondary antibodies using the ECL Western blotting detection reagents (Amersham Biosciences and GE Healthcare).

### Antibodies

The antibodies used are listed in Supplemental Table 2.

### Plasmid and oligonucleotide transfections

cDNA encoding human Trex1 with an N-terminal 3-Flag tag was amplified by PCR using the following primers: sense, 5′-gccgccATGGACTACAAAGACCATGACGGTGATTATAAAGATCATGACATCGATTACAAGGATGACGATGACAAGGGCCCTGGAGCTCGCAGACA-3′; and antisense, 5′-CTACTCCCCAGGTGTGGCCAGG-3′. The Trex1-encoding fragment was cloned into pcDNA3.1/V5-His-TOPO (Invitrogen) and fully sequenced in both directions. Plasmids were transfected into MCF7 cells using Lipofectamine LTX (Life Technologies) according to the manufacturer's instructions. Oligonucleotides were normalized to equal amounts of moles (20 pmol) in PBS for irradiation at the indicated doses before transfection.

### RNAi treatment

Cells were transfected with 20 nM siRNA oligonucleotides using HiPerFect (Qiagen) following a reverse transfection protocol. siRNA target sequences and sources are listed in Supplemental Table 3. Quantification of knockdowns for proteins was done by Fiji (ImageJ) densitometry.

### RNA extraction, reverse transcription, and real-time quantitative PCR

Real-time quantitative PCR was carried out after total RNA extraction using RNeasy kit (Qiagen) and consecutive reverse transcription to cDNA using the High-Capacity cDNA reverse transcription kit (Applied Biosystems) according to the manufacturer's instructions. Reactions were performed with the SensiMix SYBR kit (Bioline) in an Applied Biosystems 7500 real-time PCR system. All reactions were performed in triplicate. PCR mixes were pipetted by the automated pipetting system epMotion 5070 (Eppendorf). All data were normalized to β-actin and GAPDH in-house genes, and quantitative measures were obtained using the comparative CT method. Primers used for quantitative PCR are listed in Supplemental Table 4.

### Purification and labeling of cytosolic DNA

To prepare cytosolic extracts, methods were performed as described previously (Mendez and Stillman 2000) Cells were incubated with ice-cold buffer A (10 mM HEPES at pH 7.9, 10 mM KCl, 1.5 mM MgCl2, 0.34 M sucrose, 10% glycerol, 1 mM dithiothreitol, 1× Complete EDTA-free protease inhibitor cocktail [Roche]) and 0.1% Triton X-100 for 5 min on ice, and nuclei were removed by low-speed centrifugation at 1300*g* for 4 min. The supernatant was collected as soluble cytosolic fractions, while the nuclei were lysed with buffer B (3 mM EDTA, 0.2 mM EGTA, 1 mM dithiothreitol, 1× Complete EDTA-free protease inhibitor cocktail [Roche]) on ice.

Cytosolic and nuclear fractions were incubated with 100 µg/mL DNase-free RNase A (Qiagen) for 30 min at 37°C. Where indicated, samples were incubated with S1 nuclease (Thermo Fisher) for 25 min at room temperature or P1 nuclease (Sigma) for 45 min at 37°C. DNA in 10-µL aliquots were 5′ end-labeled using ddATP (Perkin Elmer) and T4 polynucleotide kinase (Thermo Fisher). Unincorporated ddATP was removed by a Micro Bio-Spin Column P-6 (Bio-Rad). The DNA-containing eluate was analyzed by nondenaturing 6% PAGE and a Typhoon PhosphorImager (Amersham Biosciences).

### Cell proliferation assay

Ninety-six-well plates were used to assess cell growth under the indicated conditions. Every condition and time point were done in triplicate. Cells were lysed directly on the plate with DNA lysis buffer (25 mM EDTA at pH 8.0, 0.1% Triton X-100) at the indicated time points. Quant-iT PicoGreen reagent (Thermo Fisher) was added, and plates were incubated for 10 min at room temperature in the dark before fluorescence with an excitation of 480 nm and an emission of 520 nm was measured with a POLARstar Omega plate reader (BMG Labtech).

### Comparative expression and survival analysis

The Metabric breast cancer data set (Curtis et al. 2012) was preprocessed, summarized, and quantile-normalized from the raw expression files generated by Illumina BeadStudio (R packages Beadarray version 2.4.2 and illuminaHuman version 3.db_1.12.2). Raw Metabric files were downloaded from European Genome–Phenome Archive (EGA; study ID EGAS00000000083). Data files of one Metabric sample were not available at the time of our analysis and were therefore excluded. The most variable Illumina probe for each gene was used as the representative of the gene's mRNA abundance levels. The probe to HGNC gene symbol mapping was performed using Ensembl BioMart version 83 (Zhang et al. 2011). Log_2_-scaled data were used for mRNA abundance analysis across breast cancer subtypes. For survival analysis, the Cox proportional hazard model was used to estimate the hazard ratio, and Wald test (R package survival version 2.38-3) was used to test the significance of outcome difference between the low- and high-expression groups. All analyses were performed in the R statistical environment (version 3.1.3).

### Flow cytometry

Cells were treated with the indicated DNA-damaging modalities and fixed in 70% ethanol for at least 30 min at −20°C before being washed with PBS and incubated with 100 µg/mL RNase A (Qiagen) for 30 min at 37°C. For staining of the DNA, 20 µg/mL propidium iodide (Sigma) was added.

Samples were analyzed using a CyAn ADP analyzer, and cell profiles were further analyzed using Flowing software (Turku Centre for Biotechnology).

### Statistical analysis

For all immunofluorescence experiments, a minimum of 100 cells was scored per time point and grouped from three independent experiments. Statistical significance was determined by the Mann-Whitney *U*-test. *P*-values for growth experiments and quantitative RT–PCR were determined using two-way ANOVA and two-tailed unpaired *t*-test, respectively (Prism 7.0a, Graphpad): *P* < 0.05 is represented by one asterisk, *P* < 0.01 is represented by two asterisks, and *P* < 0.001 is represented by three asterisks.

## Supplementary Material

Supplemental Material
