# Supplementary material for: A prosurvival DNA damage-induced cytoplasmic interferon response is mediated by end resection factors and is limited by Trex1
Source: Genes Dev. 2017 Feb 15;31(4):353–69. doi: 10.1101/gad.289769.116 (PMC5358756; doi:10.1101/gad.289769.116)
Supplement: Supplemental Material [file supp_31_4_353__index.html]

A prosurvival DNA damage-induced cytoplasmic interferon response is mediated by end resection factors and is limited by Trex1 — Supplemental Material 

# A prosurvival DNA damage-induced cytoplasmic interferon response is mediated by end resection factors and is limited by Trex1

## Supplemental Material

- Supplementary\_Figs.pdf
- SupplementaryTableErdal.pdf
- SupplementalFigLegends.docx
