# Supplementary figures and images for: A prosurvival DNA damage-induced cytoplasmic interferon response is mediated by end resection factors and is limited by Trex1
Source: Genes Dev. 2017 Feb 15;31(4):353–69. doi: 10.1101/gad.289769.116 (PMC5358756; doi:10.1101/gad.289769.116)

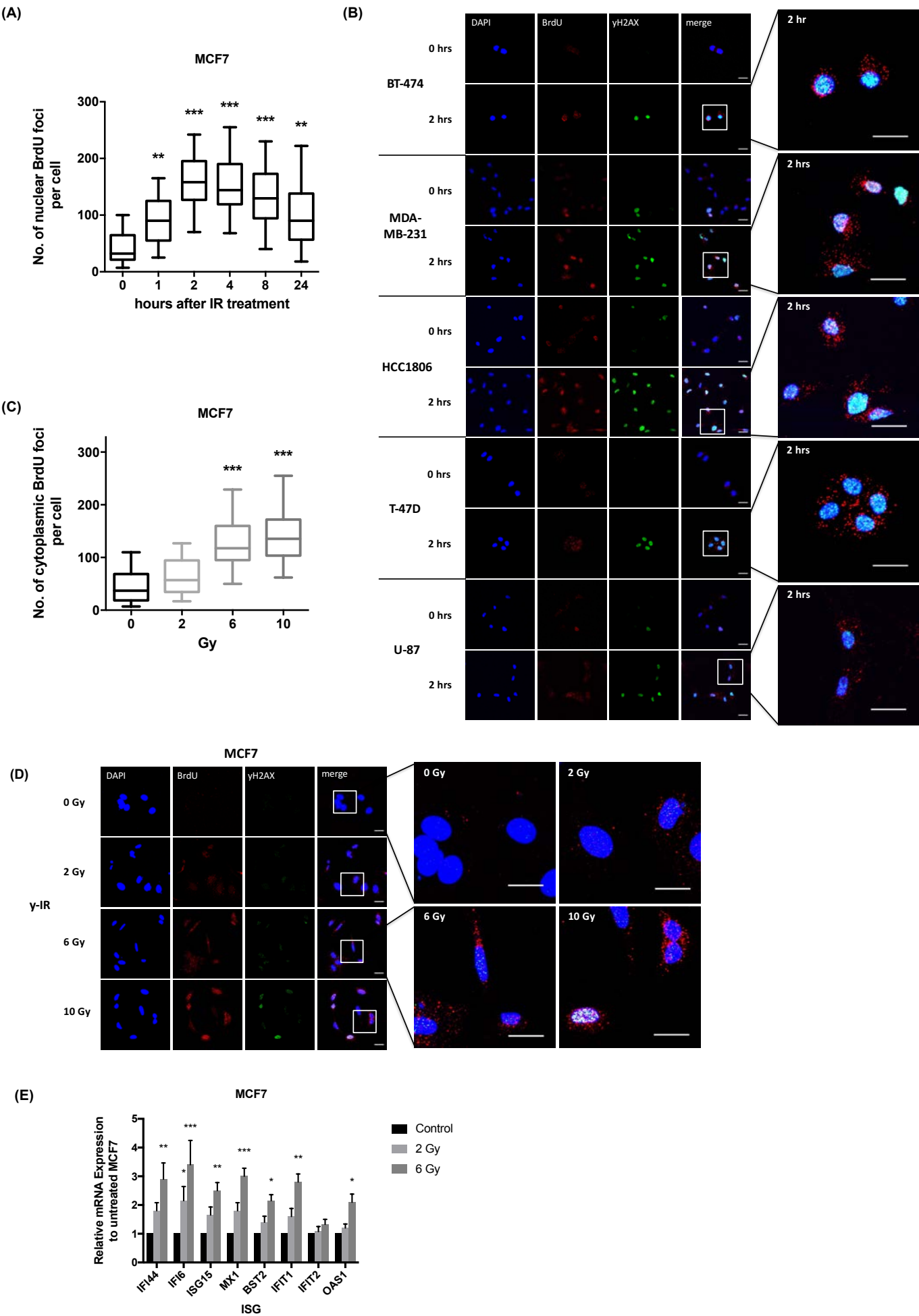

(A)

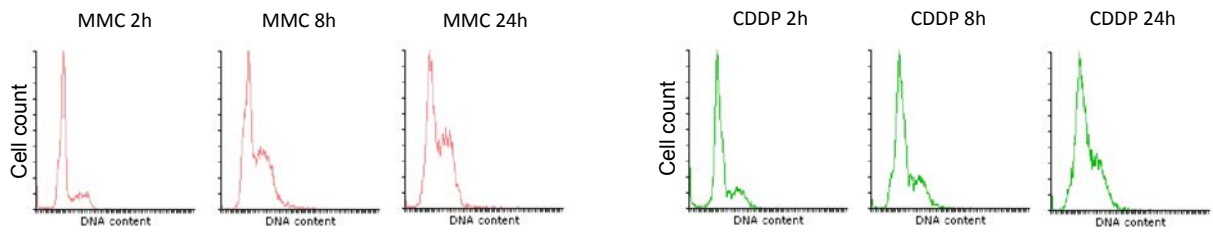

(B)

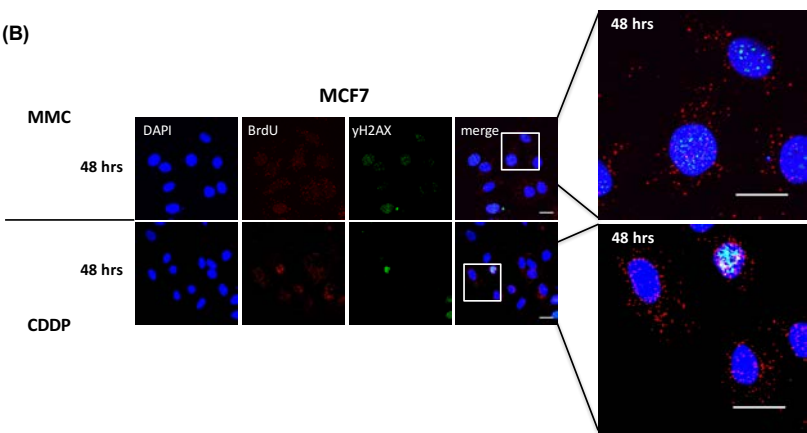

(C)

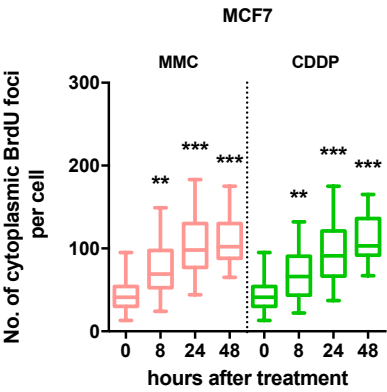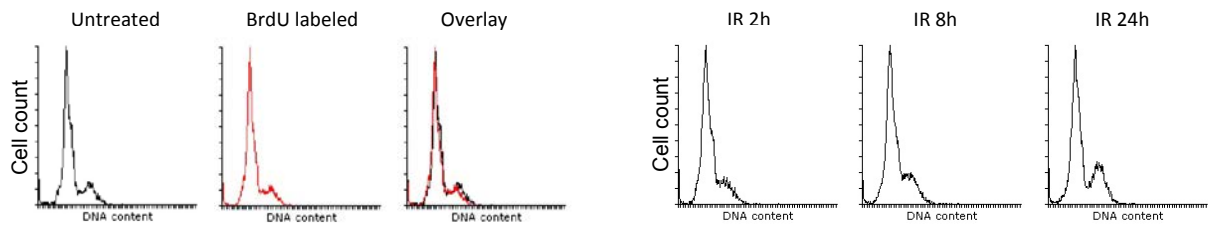

Erdal\_Supplementary Fig. 4

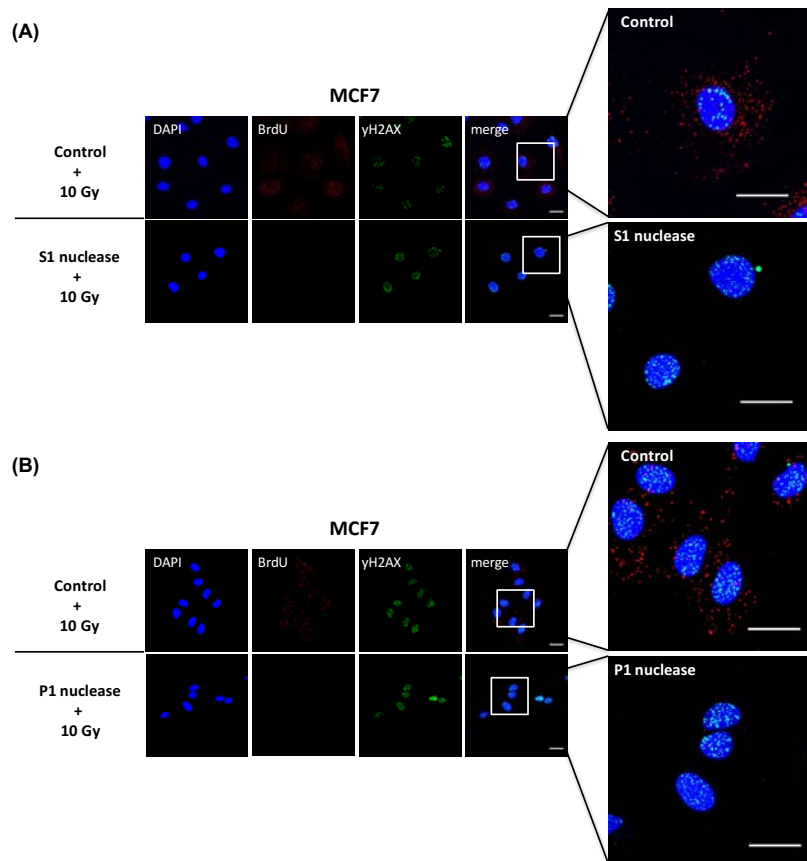

Erdal\_Supplementary Fig. 5

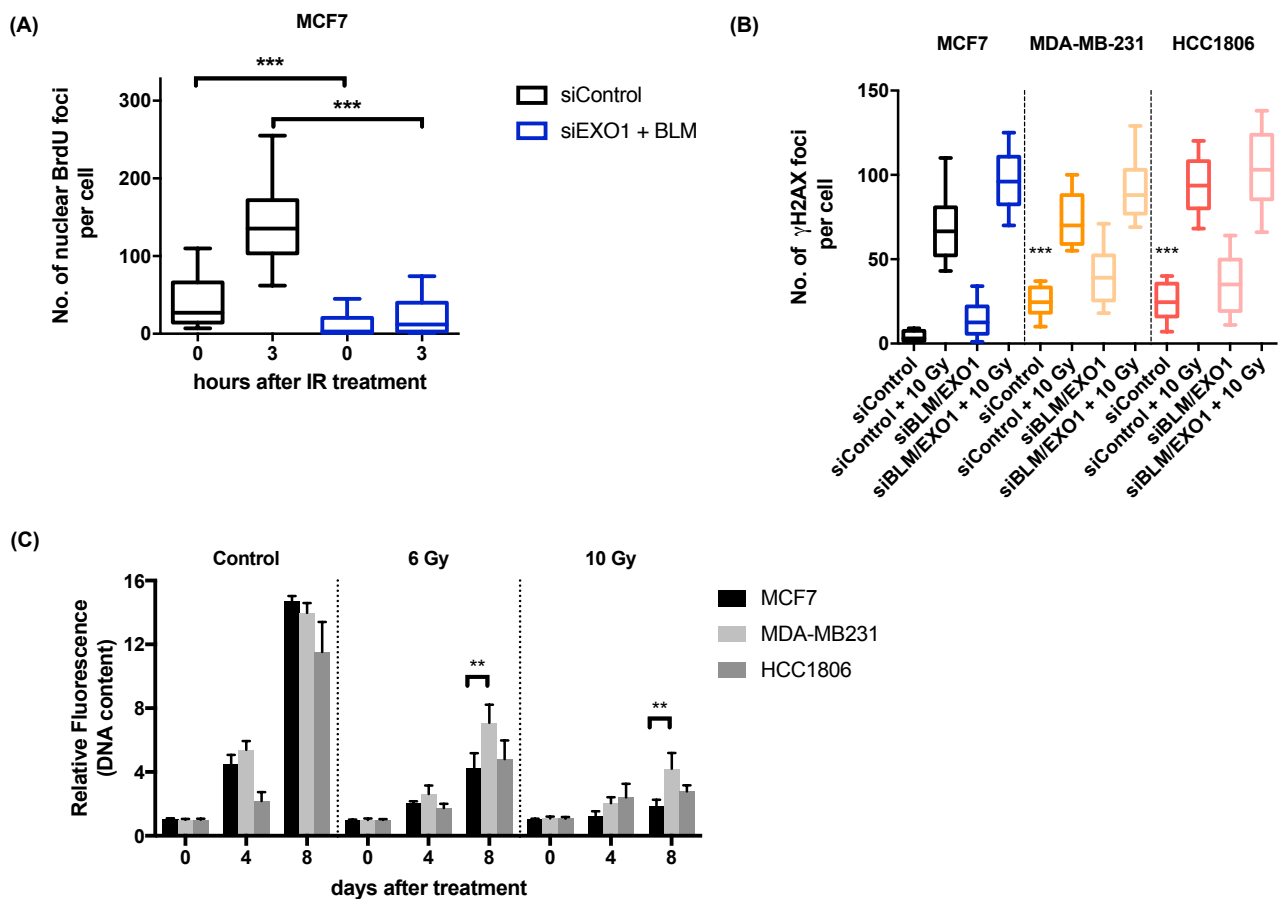

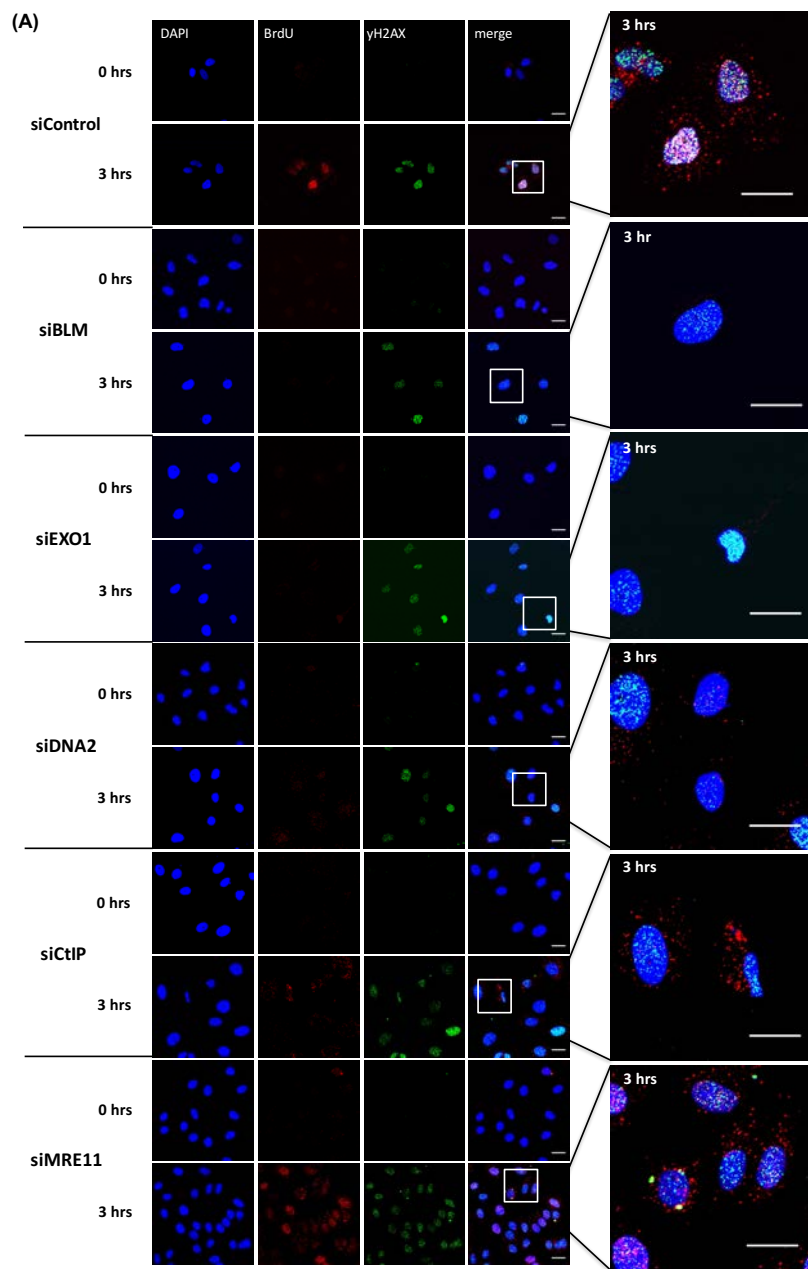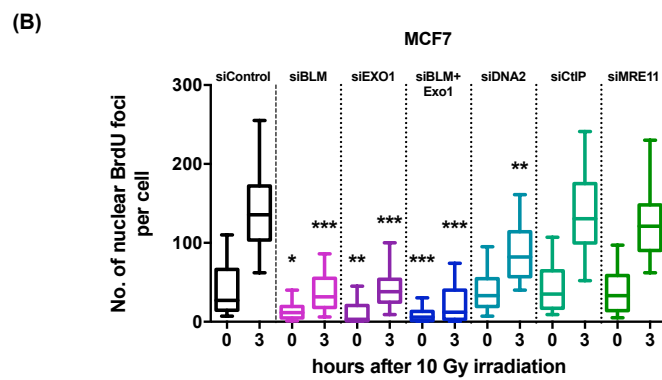

(A)

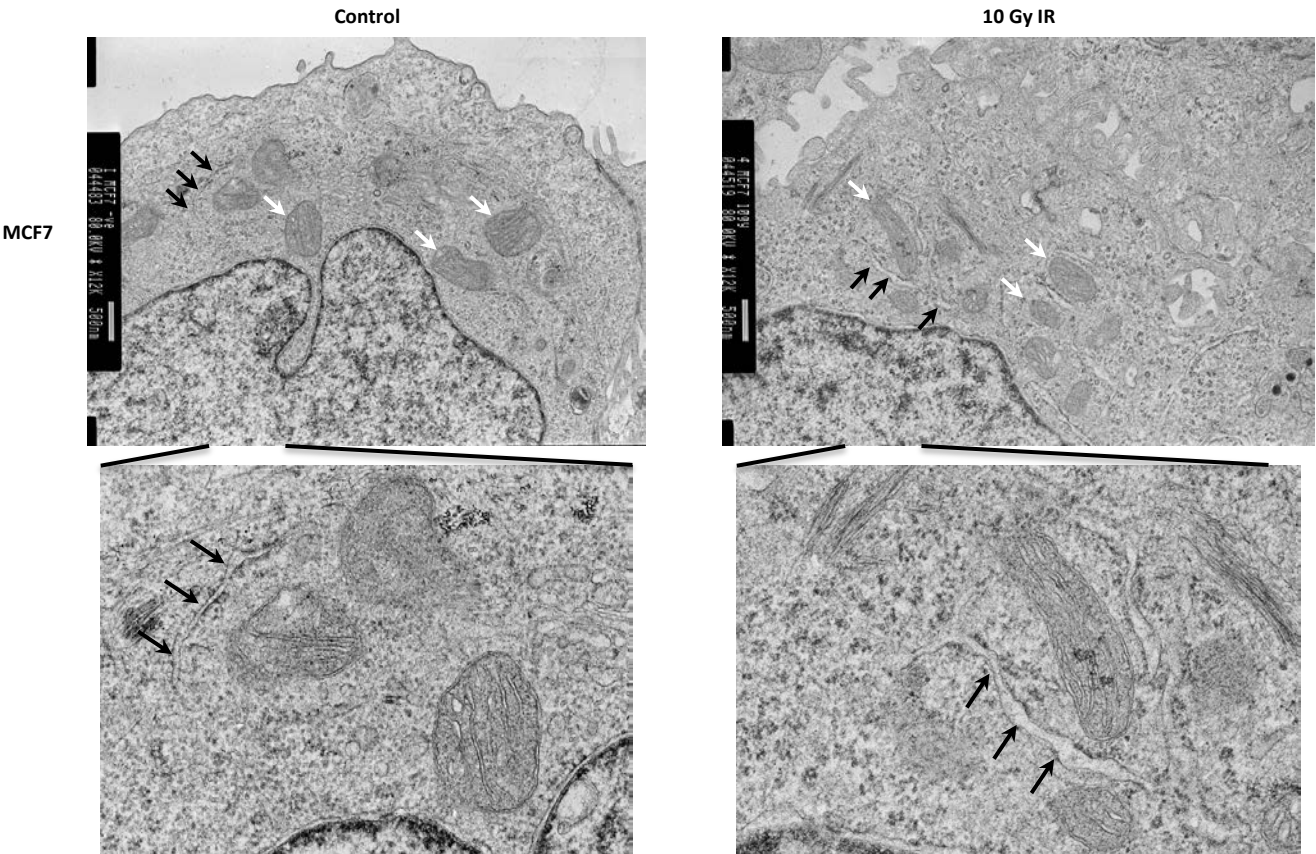

(B)

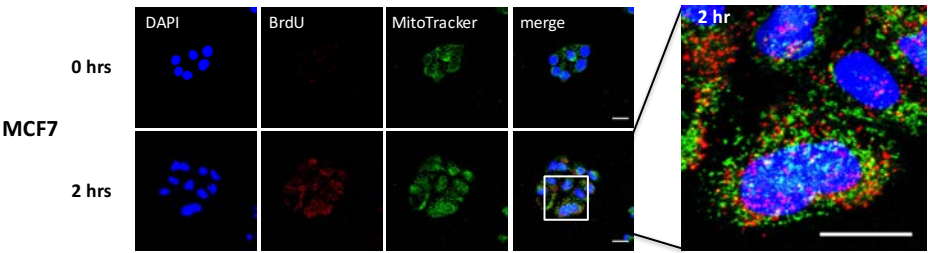

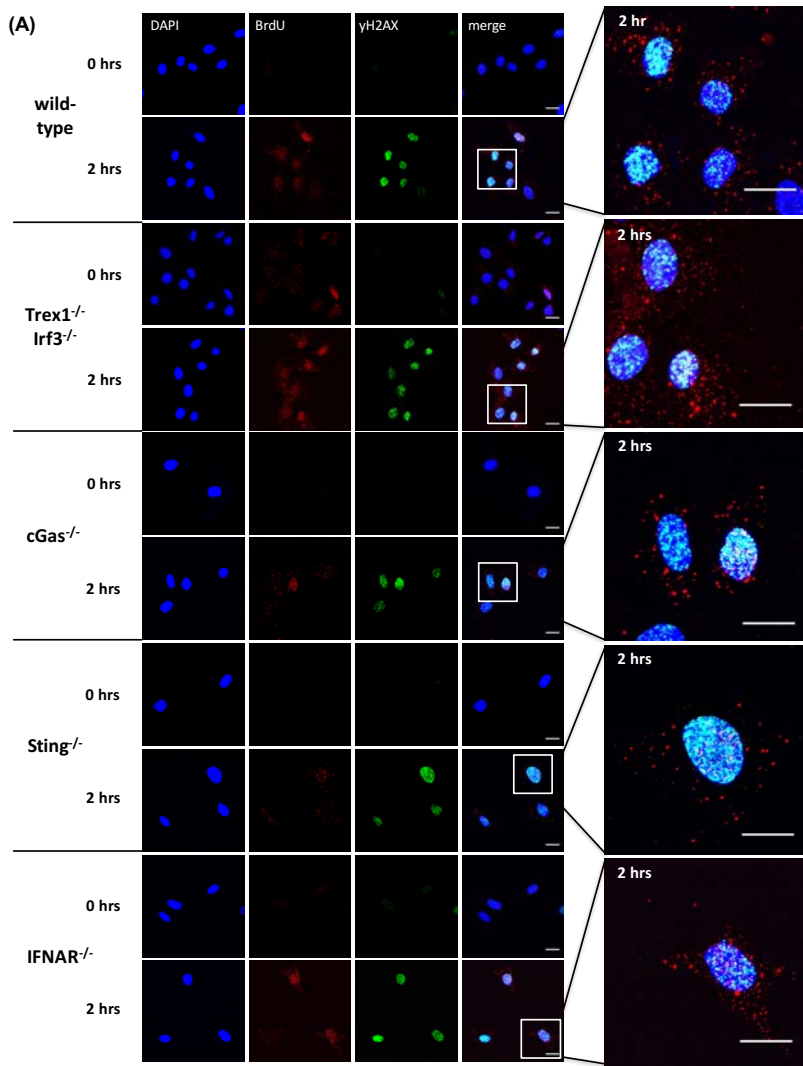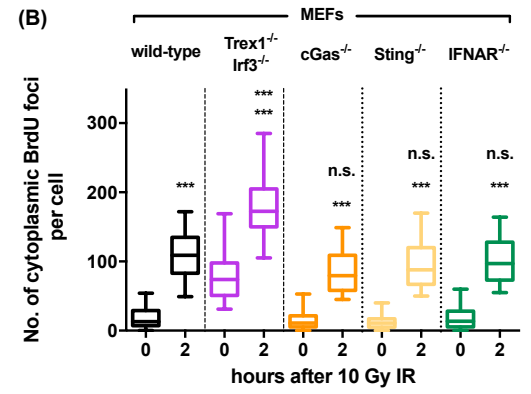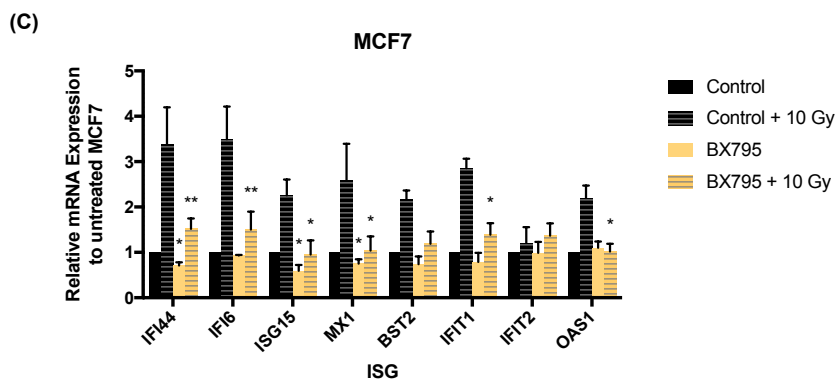

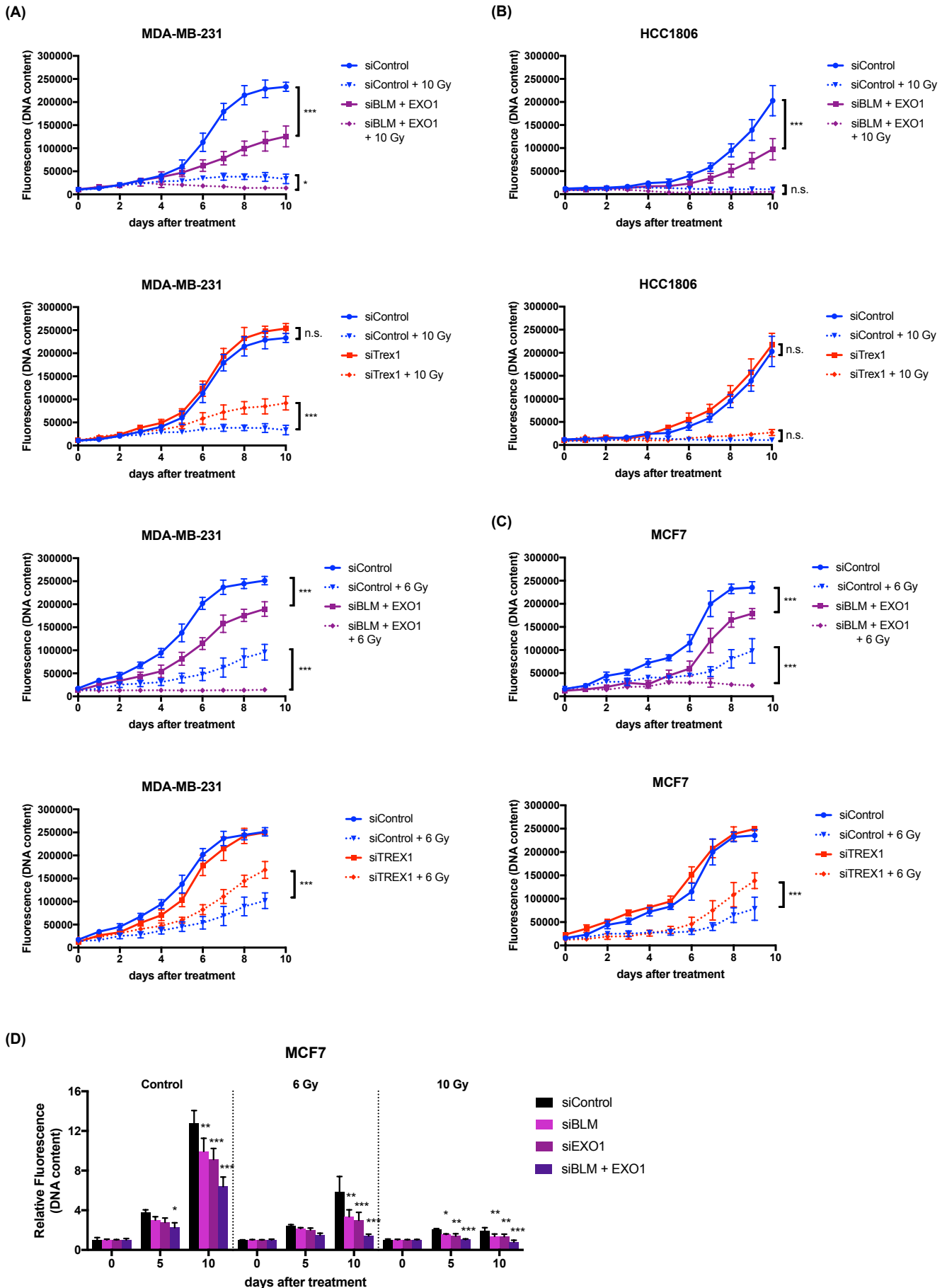

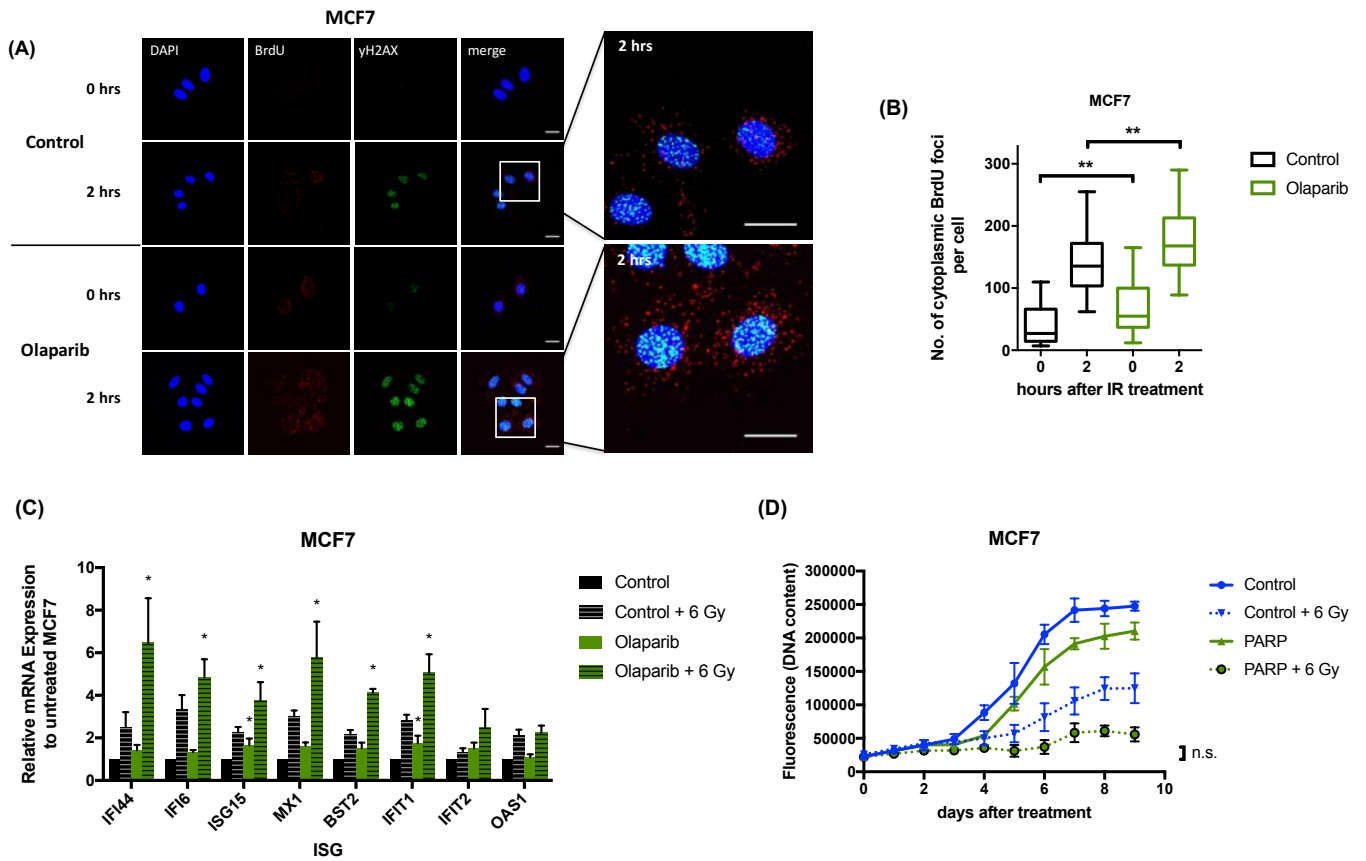

Supplement: Supplemental Material [file supp_gad.289769.116_Supplementary_Figs.pdf]
