# Supplementary material for: A prosurvival DNA damage-induced cytoplasmic interferon response is mediated by end resection factors and is limited by Trex1
Source: Genes Dev. 2017 Feb 15;31(4):353–69. doi: 10.1101/gad.289769.116 (PMC5358756; doi:10.1101/gad.289769.116)
Supplement: Supplemental Material [file supp_gad.289769.116_SupplementaryTableErdal.pdf]

Supplementary Table 1: Statistical analysis of mRNA abundance box plots

Subtypes	TREX1		BLM		EXO1		DNA2	
	FC	P	FC	P	FC	P	FC	P
Tumour (grey) vs Normal (white)	0.647	3.04E-68	0.617	6.67E-94	1.226	6.84E-117	0.147	2.28E-12
**PAM50 Subtypes (green) and Normal (white)**	**FC**	**adjP**	**FC**	**adjP**	**FC**	**adjP**	**FC**	**adjP**
Normal vs Normal-like	0.514	6.47E-11	0.330	6.47E-11	0.592	6.47E-11	0.029	0.891
Normal vs LuminalA	-0.811	6.46E-11	-0.322	6.47E-11	-0.746	6.46E-11	-0.081	0.005
Normal vs LuminalB	-0.769	6.46E-11	-0.688	6.46E-11	-1.445	6.46E-11	-0.161	3.02E-10
Normal vs Her2	-0.539	6.47E-11	-0.884	6.46E-11	-1.653	6.46E-11	-0.288	6.47E-11
Normal vs Basal	-0.263	1.18E-06	-1.139	6.46E-11	-2.031	6.46E-11	-0.241	6.47E-11
Normal-like vs LuminalA	-0.297	6.52E-11	0.008	n.s	-0.154	4.13E-03	-0.052	0.099
Normal-like vs LuminalB	-0.254	9.02E-09	-0.358	6.46E-11	-0.854	6.46E-11	-0.131	6.14E-09
Normal-like vs Her2	-0.025	n.s	-0.554	6.46E-11	-1.061	6.46E-11	-0.258	6.47E-11
Normal-like vs Basal	0.251	1.62E-07	-0.809	6.46E-11	-1.440	6.46E-11	-0.212	6.47E-11
LuminalA vs LuminalB	-0.043	n.s	0.367	6.46E-11	0.699	6.46E-11	0.080	8.44E-07
LuminalA vs Her2	0.272	6.66E-11	-0.562	6.46E-11	-0.907	6.46E-11	-0.207	6.46E-11
LuminalA vs Basal	0.548	6.46E-11	-0.817	6.46E-11	-1.285	6.46E-11	-0.160	6.47E-11
LuminalB vs Her2	0.230	4.57E-08	-0.195	1.58E-10	-0.207	1.50E-05	-0.127	2.28E - 09
LuminalB vs Basal	0.505	6.46E-11	-0.450	6.46E-11	-0.586	6.46E-11	-0.081	8.77E-05
Her2 vs Basal	0.276	7.00E-10	-0.255	6.47E-11	-0.379	6.47E-11	0.046	0.24

FC = log2 Fold change
P = P value
adjP = Adjust P value
Note: All pairwise comparisons were performed using TUKEY HSD test.

Supplementary Table 2: List of primary antibodies

Protein	Catalog No.	Clone	Manufacturer	Application	Dilution
STAT1	#9175	42H3	Cell Signaling	Western blot	1:1000
pSTAT1 (Y701)	#7649	D4A7	Cell Signaling	Western blot	1:1000
pSTAT1 (S727)	#9177	N/A	Cell Signaling	Western blot	1:1000
β-Actin	A3854	AC-15	Sigma-Aldrich	Western blot	1:10000
pChk2 (T68)	#2661	N/A	Cell Signaling	Western blot	1:1000
BLM	A300-110A	N/A	Bethyl Lab.	Western blot	1:2000
EXO1	Sc199941	N-18	Santa Cruz	Western blot	1:500
Trex1	Ab185228	EPR14985	Abcam	Western blot	1:1000
ISG15	N/A	N/A	Gift of A. Pichlmair	Western blot	1:2000
γH2AX (S139)	#9718	20E3	Cell Signaling	Immunofluorescence	1:400
BrdU	RPN202	BU-1	GE Healthcare	Immunofluorescence	1:250

Supplementary Table 3: List of siRNA target sequences

Gene		Target Sequence 5' → 3'	Source
BLM		GCTAGGAGTCTGCGTGCGATT	Invitrogen
EXO1		CAGATGTAGCACGTAATTCAA	Qiagen
DNA2 Pool	#1	TGGCGCTGGAACCTTGTCT	Invitrogen
	#2	TTTCGGTACACCTGTGCTT	Invitrogen
	#3	AAGCACAGGTGTACCGAAA	Invitrogen
	#4	AGACAAGGTTCCAGCGCCA	Invitrogen
CtIP Pool	#1	GGAGCTACCTCTAGTATCA	Eurofins
	#2	GAGGTTATATTAAGGAAGA	Eurofins
	#3	GAACAGAATAGGACTGAGT	Eurofins
	#4	GCACGTTGCCCAAAGATTC	Eurofins
MRE11 Pool	#1	GGAGGTACGTCGTTTCAGA	Eurofins
	#2	GGAAATGATACGTTTGTAA	Eurofins
	#3	CGAAATGTCACTACTAAGA	Eurofins
	#4	GAAAGGCTCTATCGAATGT	Eurofins
TREX1 Pool	#1	CCAAGACCATCTGCTGTCA	Dharmacon ON-TARGETplus
	#2	ACAATGGTGACCGCTACGA	Dharmacon ON-TARGETplus

Supplementary Table 4: List of Primers for rt-qPCR

**Human Primers**<br>**Gene**	**Forward Primer 5' → 3'**	**Reverse Primer 5' → 3'**
IFI44	ATGGCAGTGACAACTCGTTTG	TCCTGGTAACTCTCTTCTGCATA
IFI6	TCGCTGATGAGCTGGTCTGC	ATTACCTATGACGACGCTGC
ISG15	GCGAACTCATCTTTGCCAGTA	CCAGCATCTTCACCGTCAG
MX1	TTCAGCACCTGATGGCCTA	AAAGGGATGTGGCTGGAGAT
BST2	CCGTCCTGCTCGGCTTT	CCGCTCAGAACTGATGAGATCA
IFIT1	TACCTGGACAAGGTGGAGAA	GTGAGGACATGTTGGCTAGA
IFIT2	TGTGCAACCTACTGGCCTAT	TTGCCAGTCCAGAGGTGAAT
OAS1	CGCCTAGTCAAGCACTGGTA	CAGGAGCTCCAGGGCATA
DNA2	GGTGCCATACCTGTCACAAAT	AGGACCGACAAGTTTCTGTCTA
CtIP	AGATCGGTTAAGAGCAGGCTT	GATTCTGCTGCCGGATATTT
ß-actin	TGTCCACCTTCCAGCAGATGT	CACCTTCACCGTTCCAGTTTT
GAPDH	GACCCCTTCATTGACCTCAAC	CTTCTCCATGGTGGTGAAGA
**Mouse Primers**<br>**Gene**	**Forward Primer 5' → 3'**	**Reverse Primer 5' → 3'**
IFI44	AACTGACTGCTCGCAATAATGT	GTAACACAGCAATGCCTCTTGT
ISG15	GGTGTCCGTGACTAACTCCAT	TGGAAAGGGTAAGACCGTCCT
MX1	GACCATAGGGGTCTTGACCAA	AGACTTGCTCTTTCTGAAAAGCC
BST2	TGTTCGGGGTTACCTTAGTCA	GCAGGAGTTTGCCTGTGTCT
IFIT1	CCAAGTGTTCCAATGCTCCT	GGATGGAATTGCCTGCTAGA
IFIT2	AGTACAACGAGTAAGGAGTCACT	AGGCCAGTATGTTGCACATGG
ß-actin	TGTCCACCTTCCAGCAGATGT	CGCCTTCACCGTTCCAGTTTT
GAPDH	GACCCCTTCATTGACCTCAAC	CTTCTCCATGGTGGTGAAGA

Supplementary Table 5: Cell line specific duration of BrdU treatment

**Cell line**	**Hours of BrdU treatment**
MCF7	38
BT474	48
MDA-MB-231	38
HCC 1806	44
T-47D	40
U-87	38
MEFs (all)	32
