# Supplementary material for: A prosurvival DNA damage-induced cytoplasmic interferon response is mediated by end resection factors and is limited by Trex1
Source: Genes Dev. 2017 Feb 15;31(4):353–69. doi: 10.1101/gad.289769.116 (PMC5358756; doi:10.1101/gad.289769.116)
Supplement: Supplemental Material [file supp_gad.289769.116_SupplementalFigLegends.docx]

**Supplementary Figure Legends**

**Supplementary Figure 1:** *Increase of nuclear ssDNA in MCF7 and cytosolic ssDNA across different cancer cell lines*. **(a)** Quantification of nuclear BrdU foci per cell in MCF7 at the indicated time points following 10 Gy IR. Boxes represent the upper and lower quartiles, the band represents the median, and the whiskers represent minimum and maximum value in the data set. A minimum of 100 cells were scored per time point and grouped from three independent experiments. Statistical significance was determined by the Mann–Whitney U test. Stars above each box refer to indicated time point after 10 Gy treatment versus 0 hour control. **(b)** Representative confocal images of BrdU incorporated BT-474, MDA-MB-231, HCC1806, T-47D and U-87 cells at indicated timepoints after treated with 10 Gy IR. Cells stained for DAPI (blue), BrdU (red) and yH2AX (green). Scale bars, 20 µm. **(c)** Quantification of non-nuclear BrdU foci per cell in MCF7 two hours after treatment with the indicated IR doses. Boxes represent the upper and lower quartiles, the band represents the median, and the whiskers represent minimum and maximum value in the data set. A minimum of 100 cells were scored per time point and grouped from three independent experiments. Statistical significance was determined by the Mann–Whitney U test. Stars above each box refer to indicated IR dose versus 0 Gy control. **(d)** Representative confocal images of BrdU incorporated MCF7 cells two hours after treatment with indicated IR dose. Cells stained for DAPI (blue), BrdU (red) and yH2AX (green). Scale bars, 20 µm. **(e)** Relative mRNA levels of the indicated ISGs in MCF7 24 hours after treatment with 2 or 6 Gy normalized to untreated MCF7. Bars represent mean values ± SD (*n* = 3 independent experiments). A two-tailed unpaired t-test was used to determine statistical significance.

**Supplementary Figure 2:** *Cell cycle analysis and later 48 hours time point of MCF7 treated with MMC and CDDP.* **(a)** DNA content of asynchronous MCF7 cells prior and over time after treatment with 3 µM Mitomycin C and 15 µM cisplatin was determined by FACS. **(b)** Representative confocal images of BrdU incorporated MCF7 cells 48 hours after treatment with 3 µM Mitomycin C and 15 µM cisplatin. Cells stained for DAPI (blue), BrdU (red) and yH2AX (green). Scale bars, 20 µm. **(c)** Quantification of BrdU foci outside the nucleous per cell in MCF7 at indicated time points after with 3 µM Mitomycin C and 15 µM cisplatin. Boxes represent the upper and lower quartiles, the band represents the median, and the whiskers represent minimum and maximum value in the data set. A minimum of 100 cells were scored per time point and grouped from three independent experiments. Statistical significance was determined by the Mann–Whitney U test. Stars above boxes refer to indicated time point versus 0 hour control.

**Supplementary Figure 3:** *Effects of BrdU labeling and IR on cell cycle.* **(a)** DNA content of asynchronous MCF7 cells were analyzed by FACS after treatment with 10 µM BrdU over 24 hours (approximately 1.5 cell cycles) as well as following 10 Gy IR.

**Supplementary Figure 4:** *Validation of ssDNA with S1 and P1 nuclease.* Representative confocal images of BrdU incorporated MCF7 cells two hours after treated with 10 Gy IR. Prior staining cells were treated with only enzyme buffer (Control) or **(a)** S1 nuclease for 25 min at room temperature or **(b)** P1 nuclease for 45 min at 37°C. Cells stained for DAPI (blue), BrdU (red) and yH2AX (green). Scale bars, 20 µm.

**Supplementary Figure 5**: *End-resection factors are required for IR-induced accumulation of nuclear and cytosolic ssDNA.* **(a)** Quantification of BrdU foci within the nucleus per cell in MCF7 cells treated with siRNA against BLM and EXO1 at the indicated time points following 10 Gy ionizing radiation. Boxes represent the upper and lower quartiles, the band represents the median, and the whiskers represent minimum and maximum value in the data set. A minimum of 100 cells were scored per time point and grouped from three independent experiments. Statistical significance was determined by the Mann–Whitney U test. **(b)** Box-plot of yH2AX foci quantification same as in a), with MCF7, MDA-MB-231 and HCC1806 cells treated with siRNA against BLM and EXO1 after 10 Gy IR treatment. **(c)** Columns representing relative growth of MCF7, MDA-MB-231 and HCC1806 cells after 6 and 10 Gy IR treatment compared to its respective control (Day 0). PicoGreen was used to determine DNA content. Statistical significance between the data sets was determined using a two-way ANOVA test.

**Supplementary Figure 6:** *DNA end-resection factors are required for IR-induced accumulation of cytosolic ssDNA.* **(a)** Representative confocal images of BrdU incorporated MCF7 cells treated with siRNA against indicated DSB end-processing and resection factors and 10 Gy IR. Cells stained for DAPI (blue), BrdU (red) and yH2AX (green). Scale bars, 20 µm. **(b)** Quantification of BrdU foci within the nucleus per cell in MCF7 treated with siRNA against the indicated DSB end-processing and resection factors at the indicated time points following 10 Gy ionizing radiation. Boxes represent the upper and lower quartiles, the band represents the median, and the whiskers represent minimum and maximum value in the data set. A minimum of 100 cells were scored per time point and grouped from three independent experiments. Statistical significance was determined by the Mann–Whitney U test. Stars above boxes refer to the indicated time point versus matching time point of siControl.

**Supplementary Figure 7:** *Electron microscopy of MCF7 prior and after IR treatment.* **(a)** Representative electron microscopy images of MCF7 cells without treatment and 4 hours following 10 Gy IR. White arrows pointing at mitochondria. Black arrows pointing at endoplasmic reticula. **(b)** Representative confocal images of BrdU incorporated MCF7 cells at indicated timepoint after treated with 10 Gy IR and stained with MitoTracker. Cells stained for DAPI (blue), BrdU (red) and MitoTracker (green). Scale bars, 20 µm.

**Supplementary Figure 8:** *Trex1 degrades IR-induced cytosolic ssDNA.* **(a)** Representative confocal images of BrdU incorporated wild-type, Trex1 and Irf3 double knockout, *cGas^-/-^*, *Sting^-/-^* and *IFNAR^-/-^* MEF cells after 10 Gy IR. Cells were stained for DNA (DAPI, blue), BrdU (red) and yH2AX (green). Scale bars, 20 µm. **(b)** Quantification of non-nuclear BrdU foci per cell in MEFs at the indicated time points following 10 Gy IR. Boxes represent the upper and lower quartiles, the band represents the median, and the whiskers represent minimum and maximum value in the data set. Statistical significance was determined by the Mann–Whitney U test. Stars above each box refer to indicated time point after 10 Gy treatment versus 0 hour control of same cell line (bottom) and indicated time point after 10 Gy treatment versus same time point in wild-type (top). **(c)** Relative mRNA levels of the indicated ISGs after treatment with 0.5 µM BX795 (for two hours) and 24 hours following 10 Gy IR in MCF7 cells normalized to control treated MCF7. Error bars represent mean values ± SD (*n* = 3 independent experiments). A two-tailed unpaired t-test was used to determine statistical significance. Stars above columns refer to the indicated treatment versus matching treatment of control.

**Supplementary Figure 9:** *Effects of RT and BLM/EXO1 as well as Trex1 on TNBC cell lines.* **(a)** Growth curves of MDA-MB-231 depleted for BLM and EXO1 or Trex1 after treatment with 6 or 10 Gy IR. PicoGreen was used to determine DNA content. Statistical significance between the data sets was determined using a two-way ANOVA test. **(b)** Growth curves of HCC1806 depleted for BLM and EXO1 or Trex1 after treatment with 10 Gy IR. PicoGreen was used to determine DNA content. Statistical significance between the data sets was determined using a two-way ANOVA test. **(c)** Growth curves of MCF7 depleted for BLM and EXO1 or Trex1 after treatment with 6 Gy IR. PicoGreen was used to determine DNA content. Statistical significance between the data sets was determined using a two-way ANOVA test. **(d)** Columns representing relative growth of MCF7 depleted for only BLM or EXO1 and in combination of both after 6 and 10 Gy IR treatment compared to its respective control (Day 0). PicoGreen was used to determine DNA content. Statistical significance between the data sets was determined using a two-way ANOVA test.

**Supplementary Figure 10:** *Effects of PARP inhibitor Olaparib on MCF7 cells.* **(a)** Representative confocal images of BrdU-incorporated MCF7 cells at indicated time points. Cells were treated with 2.5 µM Olaparib (for 16 h) before irradiated with 6 Gy IR and subsequently stained for DNA (DAPI, blue), BrdU (red) and yH2AX (green). Scale bars, 20 µm. **(b)** Quantification of non-nuclear BrdU foci per cell in MCF7 cells treated the same as in a) at the indicated time points following. Boxes represent the upper and lower quartiles, the band represents the median, and the whiskers represent minimum and maximum value in the data set. A minimum of 100 cells were scored per time point and grouped from three independent experiments. Statistical significance was determined by the Mann–Whitney U test. **(c)** Relative mRNA levels of the indicated ISGs after treatment with 2.5 µM Olaparib and 24 hours following 6 Gy IR in MCF7 cells normalized to control treated MCF7. Error bars represent mean values ± SD (*n* = 3 independent experiments). A two-tailed unpaired t-test was used to determine statistical significance. Stars above columns refer to the indicated treatment versus matching treatment of control. **(d)** Growth curves of MCF7 treated with 2.5 µM Olaparib (for 16 h) before irradiated with 6 Gy IR. PicoGreen was used to determine DNA content. Statistical significance between the data sets was determined using a two-way ANOVA test.
